# Tubulin tyrosination regulates synaptic function and is disrupted in Alzheimer’s disease

**DOI:** 10.1093/brain/awab436

**Published:** 2022-02-11

**Authors:** Leticia Peris, Julie Parato, Xiaoyi Qu, Jean Marc Soleilhac, Fabien Lanté, Atul Kumar, Maria Elena Pero, José Martínez-Hernández, Charlotte Corrao, Giulia Falivelli, Floriane Payet, Sylvie Gory-Fauré, Christophe Bosc, Marian Blanca Ramirez, Andrew Sproul, Jacques Brocard, Benjamin Di Cara, Philippe Delagrange, Alain Buisson, Yves Goldberg, Marie Jo Moutin, Francesca Bartolini, Annie Andrieux

**Affiliations:** Univ. Grenoble Alpes, Inserm, U1216, CEA, CNRS, Grenoble Institut Neurosciences, 38000 Grenoble, France; Department of Pathology and Cell Biology, Columbia University Irving Medical Center, New York, NY 10032, USA; Department of Natural Sciences, SUNY ESC, Brooklyn, NY 11201, USA; Department of Pathology and Cell Biology, Columbia University Irving Medical Center, New York, NY 10032, USA; Univ. Grenoble Alpes, Inserm, U1216, CEA, CNRS, Grenoble Institut Neurosciences, 38000 Grenoble, France; Univ. Grenoble Alpes, Inserm, U1216, CEA, CNRS, Grenoble Institut Neurosciences, 38000 Grenoble, France; Department of Pathology and Cell Biology, Columbia University Irving Medical Center, New York, NY 10032, USA; Department of Pathology and Cell Biology, Columbia University Irving Medical Center, New York, NY 10032, USA; Department of Veterinary Medicine and Animal Production, University of Naples Federico II, 80137 Naples, Italy; Univ. Grenoble Alpes, Inserm, U1216, CEA, CNRS, Grenoble Institut Neurosciences, 38000 Grenoble, France; Univ. Grenoble Alpes, Inserm, U1216, CEA, CNRS, Grenoble Institut Neurosciences, 38000 Grenoble, France; Univ. Grenoble Alpes, Inserm, U1216, CEA, CNRS, Grenoble Institut Neurosciences, 38000 Grenoble, France; Univ. Grenoble Alpes, Inserm, U1216, CEA, CNRS, Grenoble Institut Neurosciences, 38000 Grenoble, France; Univ. Grenoble Alpes, Inserm, U1216, CEA, CNRS, Grenoble Institut Neurosciences, 38000 Grenoble, France; Univ. Grenoble Alpes, Inserm, U1216, CEA, CNRS, Grenoble Institut Neurosciences, 38000 Grenoble, France; Department of Pathology and Cell Biology, Columbia University Irving Medical Center, New York, NY 10032, USA; Department of Pathology and Cell Biology, Columbia University Irving Medical Center, New York, NY 10032, USA; Taub Institute for Research on Alzheimer's Disease and the Aging Brain, Columbia University Irving Medical Center, New York, NY 10032, USA; Univ. Grenoble Alpes, Inserm, U1216, CEA, CNRS, Grenoble Institut Neurosciences, 38000 Grenoble, France; Institut de Recherche Servier, Croissy, France; Institut de Recherche Servier, Croissy, France; Univ. Grenoble Alpes, Inserm, U1216, CEA, CNRS, Grenoble Institut Neurosciences, 38000 Grenoble, France; Univ. Grenoble Alpes, Inserm, U1216, CEA, CNRS, Grenoble Institut Neurosciences, 38000 Grenoble, France; Univ. Grenoble Alpes, Inserm, U1216, CEA, CNRS, Grenoble Institut Neurosciences, 38000 Grenoble, France; Department of Pathology and Cell Biology, Columbia University Irving Medical Center, New York, NY 10032, USA; Univ. Grenoble Alpes, Inserm, U1216, CEA, CNRS, Grenoble Institut Neurosciences, 38000 Grenoble, France

**Keywords:** tubulin, microtubule, neuron, Alzheimer’s disease, dendritic spines

## Abstract

Microtubules play fundamental roles in the maintenance of neuronal processes and in synaptic function and plasticity. While dynamic microtubules are mainly composed of tyrosinated tubulin, long-lived microtubules contain detyrosinated tubulin, suggesting that the tubulin tyrosination/detyrosination cycle is a key player in the maintenance of microtubule dynamics and neuronal homeostasis, conditions that go awry in neurodegenerative diseases. In the tyrosination/detyrosination cycle, the C-terminal tyrosine of α-tubulin is removed by tubulin carboxypeptidases and re-added by tubulin tyrosine ligase (TTL).

Here we show that *TTL* heterozygous mice exhibit decreased tyrosinated microtubules, reduced dendritic spine density and both synaptic plasticity and memory deficits. We further report decreased TTL expression in sporadic and familial Alzheimer’s disease, and reduced microtubule dynamics in human neurons harbouring the familial APP-V717I mutation. Finally, we show that synapses visited by dynamic microtubules are more resistant to oligomeric amyloid-β peptide toxicity and that expression of TTL, by restoring microtubule entry into spines, suppresses the loss of synapses induced by amyloid-β peptide.

Together, our results demonstrate that a balanced tyrosination/detyrosination tubulin cycle is necessary for the maintenance of synaptic plasticity, is protective against amyloid-β peptide-induced synaptic damage and that this balance is lost in Alzheimer’s disease, providing evidence that defective tubulin retyrosination may contribute to circuit dysfunction during neurodegeneration in Alzheimer’s disease.

## Teaser

Peris, Parato, Qu *et al*. demonstrate that tubulin tyrosine ligase (TTL) is reduced in Alzheimer’s disease and that *TTL* hemizygous mice show memory impairment and reduced synaptic integrity. Tubulin retyrosination by TTL protects against Aβ-induced synaptic damage by promoting microtubule entry into dendritic spines.

## Introduction

The neuronal microtubule cytoskeleton plays a fundamental role in the development and long-term maintenance of axons and dendrites. Research over the past two decades has revealed that dynamic microtubules, in particular, critically contribute to synaptic structure and function within both pre- and postsynaptic compartments.^1,2^ Dynamic microtubules regulate synaptic vesicle cycling by providing paths for bidirectional transport between presynaptic terminals, a rate-limiting step in exocytosis at sites of release.^3–6^ In dendritic spines, while the core cytoskeletal structure consists of actin filaments, dynamic microtubules originating in the dendritic shaft sporadically enter the spine head and directly impact on the regulation of spine composition and morphology.^7,8^ Microtubule entry into spines is dependent on synaptic activity, Ca^2+^ influx, actin polymerization and correlates with changes in synaptic strength.^9^ In cultured rodent hippocampal neurons and organotypic slices, stimulation of postsynaptic *N*-methyl-d-aspartate receptors by chemical long-term potentiation (LTP) protocols or glutamate photo-release leads to higher frequency and longer duration of spine invasions by microtubules, concurrent with spine enlargement.^10–12^ Conversely, chemical induction of long-term depression (LTD) decreases microtubule invasions, indicating that microtubules targeting into spines are sensitive to plasticity signals.^13–15^ Spine invasions, as well as synaptic plasticity, specifically involve dynamic microtubules, as both invasions and LTP are blocked when microtubule dynamics are inhibited by low doses of nocodazole^11^ or taxol.^8,16^ Consistent with these results, efficient contextual fear conditioning in mice appears to require transient accumulation of dynamic microtubules at dentate gyrus synapses.^17^ Together, these findings indicate that changes in synaptic microtubule dynamics may affect both pre- and postsynaptic functions.

Microtubule dynamics rely on the intrinsic capacity of microtubules to alternate phases of polymerization and depolymerization. Various cellular factors have been shown to modulate microtubule dynamics including the nature of tubulin isoforms, GTP hydrolysis, microtubule-associated proteins and various post-translational modifications of tubulin.^18,19^ One prominent modification is the reversible removal of the C-terminal tyrosine residue of α-tubulin subunits, which is exposed at the external surface of microtubules. This residue is cleaved off by specific tubulin carboxy-peptidases, such as the recently identified vasohibin 1 (VASH1), small vasohibin-binding protein (SVBP) and vasohibin 2 (VASH2)–SVBP complexes.^20–22^ When detyrosinated microtubules depolymerize, the tyrosine is rapidly restored on disassembled α-tubulin by the enzyme tubulin tyrosine ligase (TTL), thereby replenishing the soluble tubulin pool with full-length subunits that are then available for renewed polymerization.^23–27^ Due to these sequential reactions, tubulin undergoes a continuous cycle of detyrosination and retyrosination. Detyrosinated microtubules can be further processed by cytosolic carboxypeptidases of the deglutamylase family to generate Δ2 and Δ3 tubulins through the sequential cleavage of the final 2 or 3 amino acids, respectively.^28–31^ Δ2 tubulin cannot be re-tyrosinated by *TTL* and is thus removed from the tyrosination/detyrosination cycle.^27,32^ It follows that TTL suppression induces an accumulation of detyrosinated and Δ2-tubulins, whereas tubulin carboxypeptidase inhibition has the opposite effect.^33–35^

Newly formed tyrosinated microtubules are highly dynamic, contrary to detyrosinated microtubules that are typically more stable.^26,36,37^ Indeed, while it is known that tubulin detyrosination can occur on previously stabilized microtubules,^38,39^ there is also evidence that detyrosination of tubulin may itself promote microtubule stability by protecting microtubules from the depolymerizing activity of kinesin-13 motors.^40^ Thus, microtubule dynamics and the tyrosination/detyrosination cycle are intertwined, and modulation of the cycle is critical to processes in which microtubules need to maintain a specific dynamic state. Moreover, microtubule detyrosination confers preferential binding for specific motors and other microtubule-associated proteins, allowing tyrosination-dependent loading of selected cargos and microtubule modulators. For example, in neurons, detyrosinated microtubules play a unique role in neuronal transport by acting as preferential tracks for kinesin-1 and kinesin-2,^41–50^ while inhibiting cytoplasmic linker proteins and dynein loading onto microtubule plus ends.^33,51^ Additional roles for detyrosinated microtubules as regulators of microtubule severing enzymes have been suggested.^52,53^ In neurons, these functions regulate the trafficking of cargos, axon outgrowth and branching. For example, kinesin-1 preferentially moves along detyrosinated microtubules.^50^ Kinesin-1 is involved in mitochondria trafficking,^54^ targeting of α-amino-3-hydroxy-5-methyl-4-isoxazolepropionic acid (AMPA) receptors to dendrites^55^ and AMPA receptor-mediated synaptic transmission.^56^ Kinesin-1 may also regulate inhibitory transmission by directing the transport of gamma aminobutyric acid receptors via huntingtin-associated protein 1.^57,58^ Furthermore, robust kinesin-2 motility requires detyrosination of α-tubulin^49^ and homodimeric kinesin-2 has been implicated in the transport of glutamate receptors, whereas disruption of kinesin-2 impaired LTP, LTD and cAMP response element-binding protein responses in mice.^59^

While the function of Δ2 tubulin remains unknown, it is very abundant in neurons where it accumulates on very long-lived microtubules.^32^ Unbuffered accumulation of Δ2 tubulin, however, has been recently associated with axonal degeneration that occurs following inhibition of mitochondrial motility.^35^ In the brain, significant alteration of the tyrosination/detyrosination cycle during development modifies the relative ratio of tyrosinated, detyrosinated and Δ2 tubulin, leading to severe neurodevelopmental phenotypes in mice.^34,60^*SVBP* knock-out in mice, which leads to no activity of the tubulin carboxypeptidases VASH1 and VASH2, resulted in perturbed neuronal migration in the developing neocortex, microcephaly and cognitive defects, including mild hyperactivity, lower anxiety and impaired social behaviour.^34^ Similarly, biallelic inactivating *Svbp* variants in humans cause a syndrome involving brain anomalies with microcephaly, intellectual disability and delayed gross motor and speech development.^34,61^ Finally, *TTL* knock-out mice show disorganization of neocortical layers, disruption of the cortico-thalamic loop and death just after birth.^60,62^ However, it remains unknown whether post-developmental alteration of the tubulin tyrosination/detyrosination cycle plays a role in neurodegenerative diseases.

Alzheimer’s disease is an age-related, neurodegenerative disorder, defined by two main pathological features: overabundance of amyloid-β peptide and hyperphosphorylated tau.^63^ The most prominent clinical symptom is progressive memory loss, and decreases in synaptic density are associated with cognitive impairment.^64,65^ Alzheimer’s disease is a multifactorial disease, with both genetic and environmental aetiologies.^66^ The London (V717I) mutation in the amyloid precursor protein (APP) is sufficient to cause early onset familial Alzheimer’s disease^67^ and elevated amounts of oligomeric amyloid-β peptide (1–42) (oAβ),^68^ a variant of amyloid-β peptide more likely to oligomerize^69^ and to form disruptive plaques in the brain.^70^

Recently, increased levels of modified tubulin (polyglutamylated and/or Δ2) have been found in the hippocampi of post-mortem patients with Alzheimer’s disease, suggesting that defects in α-tubulin retyrosination may be implicated in Alzheimer’s disease.^71^ Interestingly, fluctuations of detyrosinated tubulin in synaptosomal fractions from the dentate gyrus and corresponding microtubule instability/stability phases have been associated with associative learning and memory consolidation.^17^ In that study, aged mice failed to regulate learning-dependent microtubule instability/stability phases and pharmacological disruption of either of the two phases led to deficits in memory formation. These data indicate that failure in regulating the tyrosination/detyrosination cycle occurs as a result of aging^17^ and may play a primary role in synaptic plasticity and dementia related disorders. Moreover, oAβ induces detyrosinated microtubules in hippocampal neurons and this activity contributes to tau hyperphosphorylation and tau dependent synaptotoxicity.^72^ Finally, loss of microtubule dynamics was also reported in neurons from *Kif21b* knock-out mice that exhibit learning and memory disabilities.^73^ Despite these compelling evidences, whether perturbation of the tyrosination/detyrosination tubulin cycle is a molecular driver of synaptic pathology remains unexplored.

We hypothesized that loss of tubulin retyrosination and consequential accumulation of detyrosinated and Δ2 tubulins are molecular drivers of synaptic pathology by affecting microtubule dynamics in spines. Indeed, we found that in the hippocampus of TTL heterozygous mice (*TTL^+/−^*), reduced levels of TTL expression led to significant changes in the tyrosinated/detyrosinated tubulin ratio and produced defects in synaptic transmission and plasticity that were associated with a loss of excitatory synapses. We examined whether TTL depletion was a *bona fide* feature of neurodegenerative disease and found that TTL was downregulated in both sporadic and familial Alzheimer disease, and that abnormally high levels of detyrosinated and Δ2 tubulins accumulated in brain samples of Alzheimer’s disease patients. We explored whether TTL and dynamic microtubules had a protective effect against the loss of synapses induced by oAβ. We found that microtubule entry into spines protected neurons from spine pruning and that acute oAβ exposure decreased the fraction of spines invaded by microtubules before spine loss. Remarkably, TTL expression inhibited both spine loss and the decrease in the fraction of spines invaded by microtubules, underscoring a role for retyrosinated tubulin in protecting synapses by driving dynamic microtubules into spines.

Our data unveil a role for the tyrosination/detyrosination tubulin cycle in regulating cognitive parameters such as dendritic spine density, synaptic plasticity and memory. They also provide compelling evidence for dysfunction of the cycle in Alzheimer’s disease and suggest that regulation of α-tubulin retyrosination may be critical for shielding synapses against oAβ-induced synaptic injury by promoting invasion of dynamic microtubules into spines.

## Materials and methods

### Animals

All experiments involving mice were conducted in accordance with the policy of the Institut des Neurosciences de Grenoble (GIN) and in compliance with the French legislation and European Union Directive of 22 September 2010 (2010/63/UE). Tubulin tyrosine ligase heterozygous mice (*TTL^+/−^*) were obtained as previously described^60^ and maintained in a C57BL6 genetic background by recurrent back-crosses with C57BL6 animals from Charles River Laboratories. Th1-eYFP line H mice^74^ were obtained from Jackson Labs (B6.Cg-Tgn (Thy-YFP-H) 2Jrs) and crossed with *TTL^+/−^* mice to generate a colony of C57BL6/Thy1-eYFP *TTL*^+/−^ mice. All experiments involving rats were approved by the Committee on the Ethics of Animal Experiments of Columbia University and performed according to Guide for the Care and Use of Laboratory Animals distributed by the National Institutes of Health. E18 pregnant Sprague Dawley rats were purchased from Charles River Laboratories.

### Electrophysiology

Electrophysiological tests were done with 3- and 9-month old wild-type and *TTL^+/−^* mice.

#### *Ex vivo* slice preparation

After cervical dislocation of the mice, brains were isolated and brain slices prepared from wild-type and *TTL^+/−^* male or female mice. The brain was removed quickly and 350-μm thick sagittal slices containing both cortex and hippocampus were cut in ice-cold sucrose solution (in mM: KCl 2.5, NaH_2_PO_4_ 1.25, MgSO_4_ 10, CaCl_2_ 0.5, NaHCO_3_ 26, sucrose 234 and glucose 11, saturated with 95% O_2_ and 5% CO_2_) with a Leica VT1200 blade microtome (Leica Microsystemes). After the cutting, the hippocampus was extracted from the slice and transferred in oxygenated artificial CSF (in mM: NaCl 119, KCl 2.5, NaH_2_PO_4_ 1.25, MgSO_4_ 1.3, CaCl_2_ 2.5, NaHCO_3_ 26 and glucose 11) at 37 ± 1°C for 30 min and then kept at room temperature for at least 1 h before recordings.

#### Electrophysiological recordings

Each slice was individually transferred to a submersion-type recording chamber and continuously superfused (2 ml/min) with oxygenated artificial CSF at 28°C. Extracellular recordings were obtained from the apical dendritic layers of the hippocampal CA1 area, using glass micropipettes filled with artificial CSF. Field excitatory postsynaptic potentials (fEPSPs) were evoked by the electrical stimulation of Schaeffer collaterals afferent to CA1. The magnitude of the fEPSPs was determined by measuring their slope. Signals were acquired using a double EPC 10 Amplifier (HEKA Elektronik Dr. Schulze), recorded with Patchmaster software (HEKA Elektronik Dr. Schulze) and analysed with Fitmaster software (HEKA Elektronik Dr. Schulze). Input/output (I/O) curves characterizing basal glutamatergic transmission at CA3-CA1 synapses of wild-type and *TTL^+/−^* mice were constructed by plotting mean fEPSPs slopes ± standard error of the mean (SEM) as a function of stimulation intensity (10 to 100 μA). For LTP experiments test stimuli were delivered once every 15 s and the stimulus intensity was adjusted to produce 40–50% of the maximal response. LTP was induced using a theta-burst stimulation (theta-burst stimulation involved five trains with 10 bursts of four pulses delivered at 100 Hz, an interburst interval of 200 ms and 20-s interval between each train). The average value of the fEPSP slope was expressed as a percentage of the baseline response ± SEM.

### Behavioural studies

Behavioural tests were done in 3–4-month-old wild-type and *TTL^+/−^* mice. Evaluation of cognitive function was performed with spontaneous alternation in the Y-maze test for working memory, and with the novel object recognition test for episodic memory. Procedures were performed during the animals’ light cycle. For each test, animals were habituated to the test room for 30 min; room lighting was set to 150 lx and ambient sound was provided by white noise generators set for 60 dB of white noise. Animal testing order within a test was organized to prevent animals from being single housed immediately before being tested. Experimenter was blinded to the genotype of animals during testing. Only males were used.

#### Spontaneous alternation test

Spontaneous alternation tests were conducted in a Y-shaped maze, made of black Plexiglas. The maze was heightened to ∼1 m high, and comprised three arms of equivalent size (length = 38 cm; width = 8 cm; height of walls = 15 cm), numbered from 1 to 3, and by equivalent angles between them (120°). The mouse was put in the centre of the maze, the nose in the direction of the bottom of one of the arms. The mouse was free to explore the environment for 5 min. The experimenter observed the behaviour by using a camera located in an independent room and noted the sequence of successive arm visits. A visit or an entrance into an arm was defined as four legs in the zone of the arm. The apparatus was cleaned with alcohol and subsequently with water between each mouse. An alternation was defined as a visit in a given arm followed by a visit into another arm. The successive sequence of visits during 5 min determined the level of alternation. The performance of the animal was estimated by calculating a percentage of alternation: alternation index = [number of alternations / (total number of visited zones − 2)] × 100.

#### Novel object recognition test

Novel object recognition tests were performed in a Y-shaped maze, to about 1 m in height, consisting of three opaque black plastic arms of equal size (length = 38 cm, width = 8 cm, height of wall = 15 cm), numbered 1–3 and at a 120° angle from each other. Four different objects by size, shape and pattern were used. The recognition test had three phases: habituation, familiarization and recognition. For habituation at Day 1, the mouse was placed in the centre of the Y-maze, without object, to freely explore the three arms for 10 min. For familiarization at Day 2, the mouse was again placed in the centre of the Y-maze which contained at each end different objects. The mouse freely explored for 5 min, during which it can familiarize with these three objects. For recognition test, 1 h after familiarization, the mouse was placed in the centre of the Y-maze where one object presented during the familiarization phase was replaced by a new object. The mouse freely explored for 5 min and the experimenter measured the time of exploration of each object using a semi-automatic key. The assessment criterion was the difference between the time of exploration of the new object and the mean time of the time of exploration of the two familiar objects: recognition index = difference [new object − (mean of the two familiar objects)] durations (in seconds) of exploration.

### Plasmids

For lentiviral experiments, vector eGFP-pWPT (Addgene no. 12255, kind gift from D. Trono) was used to express eGFP, and cDNA encoding human *TTL* (NP_714923, Origene no. RC207805L2) was cloned in it for TTL expression. PCR ampliﬁcation and cloning of *TTL* cDNA were performed with Phusion DNA polymerase (Thermo Scientiﬁc) and In-Fusion HD Cloning kit (Clontech), respectively. *eGFP* cDNA was removed during the cloning process to produce an untagged TTL. For lentiviral shRNA expression, two *TTL* shRNA sequences, cloned in pLKO.1 vector, were purchased from Sigma-Aldrich: shTTL1 (TRCN0000191515, sequence: 5′-CCG GCA TTC AGA AA GAG TAC TCA ACT CGA GTT GAC TAC TCT TTC TGA ATG CTT TTT TG-3′) and shTTL2 (TRCN0000191227, sequence: 5′-CCG GCT CAA AGA ACT ATG GGA AAT ACT CGA GTA TTT CCC ATA GTT CTT TGA GTT TTT TG-3′).^35^ The SHC001 pLKO.1-puro empty Vector (Sigma) was used as control (shControl). For the transfection experiments, the plasmid encoding pCMV-*EB3-EGFP* was a kind gift from Dr Frank Polleux.^72^ Kind gifts from Dr Erik Dent include the plasmids *EB3-tdTomato* (Addgene no. 50708) and the plasmid encoding *DsRed2* (Clontech), cloned into a pCAX vector. The plasmid pEGFP-N1 with a CMV promoter was also used (Addgene no. 6085-1). All constructs were veriﬁed by sequencing (Euroﬁns and Genewiz). Plasmids were purified with HiPure Plasmid Maxiprep kits (Invitrogen).

### Amyloid-β peptide (1–42) oligomer preparation

Oligomer-enriched preparations of amyloid-β peptides (1–42) were obtained according to previously published methods.^72^ Briefly, the lyophilized amyloid-β peptide (1–42) (rPeptide) was resuspended in 1,1,1,3,3,3-hexafluoro-2-propanol to a concentration of 1 mM and monomeric amyloid-β peptide (1–42) aliquots were resuspended in anhydrous dimethyl sulphoxide to 5 mM followed by vortexing and 10-min sonication. The resuspended peptide was diluted to 100 µM in ice-cold Ham’s F-12 medium and incubated at 4°C for 24 h before use.

### Lentivirus production

Lentiviral particles were produced using the second-generation packaging system as previously described.^72,75^ Lentivirus encoding *GFP* or *TTL* cDNA (packaging vectors, pWPT-based vector, Addgene) and shTTL1, shTTL2 and control shRNA (packaging vectors pLP1, pLP2 and pLP-VSV-G, Thermofisher) were produced by cotransfection with the psPAX2 and pCMV-VSV-G helper plasmids, into human embryonic kidney 293T cells obtained from ATCC (ATCC-CRL-3216) using the calcium phosphate transfection method. Viral particles were collected 48 h after transfection by ultra-speed centrifugation, before aliquoting and storage at −80°C.

### Primary hippocampal neuronal cultures

#### Mouse hippocampi

Mouse hippocampi (E18.5) were digested in 0.25% trypsin in Hanks’ balanced salt solution (HBSS, Invitrogen, France) at 37°C for 15 min. After manual dissociation, cells were plated at a concentration of 5000–15 000 cells/cm^2^ on 1 mg/ml poly-l-lysine-coated coverslips for ﬁxed samples, or on ibidi glass bottom µDishes (35 mm) for live imaging. Neurons were incubated 2 h in DMEM-10% horse serum and then changed to MACS neuro medium (Miltenyi Biotec) with B27 supplement (Invitrogen).

#### Rat hippocampi

Rat hippocampi were dissected from E18 embryos, and neurons were plated on 100 µg/ml poly-d-lysine–coated 12-well plates at the density of 3 × 10^5^ cells/well for biochemistry assays, 5 × 10^4^ cells/dish for live imaging in the chamber of 35-mm MatTek dishes or 4 × 10^4^ cells/coverslip on 18-mm coverslips for fixed samples. Primary neurons were maintained in Neurobasal medium (Invitrogen) with the supplement of 2% B-27 (Invitrogen) and 0.5 mM glutamine (Invitrogen) and one-third of the medium was changed every 3–4 days up to 4 weeks in culture.

#### Lentivirus infection

To perform dendritic spine quantification in cultured mouse neurons, 1/100 of a hippocampal cell suspension was infected by 15-min incubation with GFP lentivirus (Lv) at a multiplicity of infection of 40. The infected population was then mixed with non-transduced cells before plating. Some of those cultures were infected at 1 day *in vitro* (DIV) with TTL lentivirus at a multiplicity of infection of 5. Hippocampal neurons were incubated for 18 DIV at 37°C, 5% CO_2_ in a humidiﬁed incubator and then ﬁxed with 4% paraformaldehyde in 4% sucrose-containing phosphate-buffered saline (PBS) for 20 min. To induce acute TTL reduction, hippocampal neurons from wild-type rat embryos were infected at DIV 14 or DIV 17 with lentiviral vectors containing either control or 1 of 2 independent tubulin tyrosine ligase-targeting shRNAs and incubated until DIV 21. Ectopic expression of *TTL* for microtubule spine dynamics experiments was also achieved through lentiviral infection, with infection again occurring at DIV 14 and incubation until DIV 21.

### Imaging of dendritic spines

For *in vivo* ﬁxed samples, serial sections were obtained from cortical layer V of 3-month-old Thy1eYFP-H wild-type and Thy1eYFP-H *TTL^+/−^* male mice brains. Briefly, mice were anaesthetized, perfused transcardially with saline followed by 4% paraformaldehyde and brain recovered. For cultured samples, hippocampal neurons from wild-type, *TTL^+/−^* and TTL^−/−^ embryos were infected with eGFP containing lentivirus and fixed at DIV 18. Dendritic segments visualized by soluble eYFP and eGFP, respectively, were obtained using a confocal laser scanning microscope (Zeiss, LSM 710). Serial optical sections (1024 × 1024 pixels) with pixel dimensions of 0.083 × 0.083 μm were collected at 200-nm intervals, using a ×63 oil-immersion objective (NA 1.4). The confocal stacks were then deconvolved with AutoDeblur. For *in vitro* analysis of spines in cultured hippocampal neurons isolated from rat embryos and infected with TTL-targeting shRNAs, DiOlistic labelling using the Helios gene gun system (Bio-Rad) was performed according to the manufacturer’s instructions. Tungsten particles (1.1 μm, Bio-Rad) coated with DiI (Invitrogen), which defines the neuronal architecture in red, were delivered into hippocampal neurons fixed in 4% paraformaldehyde prior to mounting with ProLong Gold antifade mounting reagent (Invitrogen). Neurons were imaged the next day using an Olympus IX8Andor Revolution XD Spinning Disc Confocal System. *Z*-stack images were taken at 0.2-µm step lengths for 10–15 stacks and shown as maximum projections. Dendritic spine analysis (spine counting and shape classiﬁcation) was performed on the deconvolved stacks using Neuronstudio and Neurolucida 360.^76^ All spine measurements were performed in 3D from the *z*-stacks. The linear density was calculated by dividing the total number of spines present on assayed dendritic segments by the total length of the segments. At least three dendritic regions of interest were analysed per cell from at least three independent cultures in each experimental condition.

### Live imaging of microtubule dynamics at spines

Rat neurons grown on 35 mm glass bottom live imaging dishes (MaTek) were cotransfected with plasmids encoding either EB3-eGFP and DsRed or EB3-tdTomato and eGFP using Lipofectamine 2000 (Invitrogen). Live cell imaging was performed 24–48 h after transfection in complete HBSS media (HBSS, 30 mM glucose, 1 mM CaCl_2_, 1 mM MgSO_4_, 4 mM NaHCO_3_ and 2.5 mM HEPES, pH 7.4) using an IX83 Andor Revolution XD Spinning Disc Confocal System. The microscope was equipped with a ×100/1.49 oil UApo objective, a multi-axis stage controller (ASI MS-2000) and a controlled temperature and CO_2_ incubator. Movies were acquired with an Andor iXon Ultra EMCCD camera and Andor iQ v.3.6.2 live cell imaging software. Movies of microtubule dynamics at spines were acquired at 4 s/frame for 10 min with three *z*-stack planes at 0.4-µm step size. Maximum projections of movies were performed by Image Math within Andor software, exported as Tiff files and analysed in ImageJ. Kymographs were generated by drawing a region from the base of the spine to the tip of spine head. Parameters describing microtubule invading into spines were defined as follows: percentage of spines invaded 10 min^−1^ were the number of spines invaded by microtubules during 10-min movie/total number of spines in the imaging field and the invasion lifetime was the total duration of EB3 residing in a spine including comet lifetimes of multiple invasions.^10^ Parameters describing microtubule dynamics were defined as follows: rescue/nucleation frequency was the number of rescue or nucleation events per μm^2^ per min; catastrophe frequency was the number of full tracks/total duration of growth; comet density was the number of comets per μm^2^ per min; growth length was the comet movement length in μm; comet lifetime was the duration of growth and growth rate was the growth length/comet lifetime.^77^

### Analysis of spine structural plasticity

Morphologies (stubby, mushroom, thin) of all protrusions invaded or not invaded by EB3 in the same imaging field before (0 h) and after vehicle or oAβ treatment (2 h) were individually documented using NeuronStudio Software. Percentages of the same protrusions changing to pruned, thin, mushroom or stubby spines were then calculated based on total number of spines invaded or not invaded by EB3 in the same field. *χ*^2^ tests were performed on spine persistence or pruning in vehicle and oAβ treated neurons at 0 and 2 h. *χ*^2^ tests were also performed on spine morphology changes (to thin, to stubby, to mushroom, to pruned) in vehicle and oAβ-treated neurons at 0 and 2 h.

### Biochemical analysis of post-mortem human brain tissues

Human brains were provided by the Human Brain Tissue Bank, Semmelweis University, Budapest, Hungary. Tissue samples consist of four regions of brain (entorhinal cortex, hippocampus, temporal and lateral prefrontal cortex) coming from a panel of 29 male and female patients aged from 52 to 93 years: 11 controls, 5, 6 and 7 from each group corresponding to Braak stadium I–II, III–IV and IV–V (Supplementary Table 1).

#### Extraction

Brain samples were homogenized 2 × 30 s at room temperature in (10% vol/w) 10 mM Tris, 0.32 M sucrose, pH 7.4 containing complete inhibitors cocktail (Roche) using ready to use Precellys Lysing Kit (Bertin Technologies) in a Minilys apparatus. After lysis, the homogenates were collected, frozen in liquid nitrogen and then stored at −80°C until use. When needed, frozen aliquots were diluted vol/vol with RIPA buffer (50 mM Tris, 150 mM NaCl, 1% NP40, 0.5% deoxycholate, 0.1% SDS, pH 8) stirred 30 min at 4°C and then centrifuged 10 min at 14 000*g* at 4°C. Supernatants were frozen in liquid nitrogen and then stored at −80°C until use.

#### Antibodies

Monoclonal rat anti-tyr-tubulin (YL1/2), polyclonal anti-detyrosinated, Δ2 tubulin antibodies and monoclonal anti α tubulin antibody (α3A1) were produced in the Andrieux’s laboratory as previously described.^32^ Mouse monoclonal anti-TTL antibody ID3 was as described^78^ and polyclonal antibody 13618-1-AP was purchased from Proteintech.

#### Western blot analysis and quantification

RIPA supernatants (10 µl) were subjected to electrophoresis on stain free 4–15% gels (Bio-Rad) and then quickly transferred to nitrocellulose using Trans-Blot Turbo Transfer System (Bio-Rad). Proteins on the membrane were revealed using specific antibodies against different forms of modified tubulin (tyrosinated, detyrosinated, Δ2) and α tubulin. Anti-Tyr-Tub (1/10 000), anti-deTyr-Tub (1/20 000), anti Δ2-Tub (1/20 000) and anti α tubulin (1/10 000) antibodies were used with the appropriate peroxidase-/labelled secondary antibodies. Secondary antibody signal was revealed using Pierce ECL western blotting substrate (Thermo Scientific) and analysed with ChemiDoc™MP Imaging System (Bio-Rad) using Image Lab software (stain free gel protocol) for quantification. For each lane of the blot, the software measures the integrated volume of the band corresponding to the antigen of interest. The signal is then normalized according to the total protein measured in the same lane. For every blot, one lane is dedicated to an internal standard corresponding to a wild-type sample (used for the entire study) and the protein-normalized signal of this standard is considered as 100%, therefore each unknown sample is calculated as a percentage of this standard. For each brain sample, three independent blots were performed and the mean intensity was calculated.

#### ELISA

The assay was routinely performed in high binding 96-well plates (Immulon 4 HBX, Thermo Fisher). Washings throughout the assay were: 200 μl/well, three times per washing step with PBS buffer solution containing 0.05% Tween 20 (PBST). Anti-TTL antibody ID3 was coated at 1/2000 in PBS (100 μl/well) overnight (∼16 h) at 4°C. After washing, the plates were blocked by adding 2% Bovine Serum Albumin (BSA) in PBS (200 μl/well) for 6 h at room temperature. The plates were then washed and incubated overnight (∼16 h) at 4°C with TTL standards or brain samples diluted in 1% BSA in PBS (100 μl/well). The sample diluent served as negative control. Washed plates were then incubated for 1 h at room temperature with anti-TTL antibody (13618-1-AP) at 1/2000 in 1% BSA in PBST (100 μl/well). Washed plates were incubated for 1 h at room temperature with peroxidase rabbit antibody diluted 1:10 000 in BSA/PBST (100 μl/well). The plates were washed and incubated with 3,3′,5,5′-tetramethylbenzidine Liquid Substrate (Sigma-Aldrich) (100 μl/well). Reaction was stopped after 5 min by adding Stop Reagent (Sigma-Aldrich) (100 μl/well). Absorption was determined at 450 nm on Pherastar FS (BMG Labtech). For each brain sample, three independent ELISA were performed and the mean value was calculated. Purified TTL was used for normalization (kind gift from M. Steinmetz).^27^

### Biochemical analysis of cultured primary neurons

Cortical neurons (17 DIV) isolated from mouse embryos were transduced or not with a lentivirus expressing *TTL* and treated with DMSO or with 100 nM oAβ (48 h) before collection, washing with phosphate-buffered saline medium at 37°C and lysis in Laemmli buffer. The protein contents of TTL, tyrosinated and detyrosinated tubulin were analysed by quantitative western blot with the protocol used for human brain samples as described previously. Several neuronal cultures were used as indicated in figure legends and for each sample, three independent blots were performed.

### Biochemical analysis of mouse brain tissues

Mice hippocampi were homogenized in a lysis buffer (PBS) without CaCl_2_ and MgCl_2_, 14190-094 Life Technologies) supplemented with protease (P8340, Sigma) and phosphatase inhibitor cocktails (P5726 and P0044, Sigma) at 150 mg/ml, using a Precellys apparatus homogenizer (2 × 20 s, 5000 rpm). Lysates were then centrifuged at 21 000*g* for 20 min at 4°C. The resulting supernatants were collected and protein concentrations were determined using bicinchoninic acid assays (Pierce/Thermo Fisher Scientific). Samples were stored at −80°C until analysis.

#### Automated western blotting

Automated western blotting was performed with equal concentrations of protein per sample (0.125 µg/µl) using the Peggy Sue™ system (Protein Simple) according to the manufacturer’s instructions. Detection of tyrosinated, detyrosinated tubulins and TTL levels were assessed using appropriate primary antibodies as detailed previously for human samples. Data were analysed with Compass software (Protein Simple).

### Immunohistochemical analysis of post-mortem brain tissues

De-identified human autopsy brain tissue was obtained from the New York Brain Bank at Columbia University (New York, NY, USA). Neuropathologically confirmed Alzheimer’s disease cases and controls were processed following published protocols.^79^

#### Antibodies

Antibodies anti-Δ2 tubulin (AB3203) was from Millipore, anti-detyrosinated tubulin (MAB5566) from Sigma-Aldrich and anti-tau AT8 (MN1020) from Invitrogen.

#### Immunolabelling

Immunolabelling brain paraffin blocks were cut into 5 μm sections and deparaffinized in xylene (7 min twice) followed by 95% ethanol, 90% ethanol, 80% ethanol and 70% ethanol (5 min each). After washing the slices in distilled H_2_O three times, citric acid was used to retrieve antigen by boiling samples for 15 min. Sections were cooled for 15 min, washed three times with PBS and blocked with serum for 1 h at room temperature before staining with primary antibodies (anti-detyrosinated tubulin, 1/100; anti Δ2 tubulin 1/500 and AT8 anti-Tau, 1/500) at 4°C overnight. The next morning sections were washed three times with PBS and stained with appropriate secondary antibodies (Cy3 donkey anti-mouse, 1/200; Alexa 488 donkey anti-rabbit, 1/200; DAPI, 1/1000) for 1 h at room temperature. Stained samples were washed three times with PBS and incubated in 0.1% black Sudan in 70% ethanol for 5 min to reduce auto-fluorescence of lipofuscin, rinsed with 70% ethanol until the black was gone and then it was rehydrated in distilled H_2_O.

#### Image acquisition and analyses

Coverslips were mounted with Fluoromount before imaging using an Oympus VS-ASW FL 2.7 (Build 11032) slide scanner and Olympus soft imaging solutions camera XM10. Images were taken using a ×10 objective and same exposure time was used for the same primary antibody (detyrosinated tubulin: 100 ms; Δ2: 200 ms; AT8 tau: 10 ms; 4′,6-diamidino-2-phenylindole: 10 ms). The images were converted into Tiff files for analysis using MetaMorph software. Pyramidal neuron cell bodies and proximal dendrites were randomly selected in the anterior hippocampal formation and average fluorescence intensity was measured for detyrosinated and Δ2 tubulins, as well as for AT8. An average of 150 neurons were selected for each case. Pyramidal neurons were arbitrarily classified into low AT8 (1-300 A.U.), intermediate AT8 (300.01-1000 A.U.) and high AT8 (1000.01-2400 A.U.) based on AT8 staining intensity in the cell body.

### Mutant APP and isogenic control iPSC cell maintenance and differentiation

Human induced pluripotent stem cells (iPSCs) in which the *APPV717I* (London) mutation was knocked into one allele of the control IMR90 cl.4 iPSC line (WiCell)^80–82^ using CRISPR–Cas9 was generated by the laboratory of A.S., as has been described previously.^83^

#### Maintenance

Maintenance APPLon knock-in (cl. 88) and the isogenic parent line were maintained feeder-free in StemFlex media (Life) and Cultrex substrate (Biotechne).

#### Neuronal differentiation

Neuronal differentiation bankable neural progenitors were first generated using manual rosette selection and maintained on Matrigel (Corning) as has been described previously.^83,84^ Terminal differentiations were carried out by plating 165 000–185 000 neural progenitor cells per 12-well plate in N2/B27 media (DMEM/F12 base) supplemented with brain-derived neurotrophic factor (20 ng/ml; Biotechne) and laminin (1 µg/ml; Biotechne) on PEI (0.1%; Sigma)/laminin (20 µg/ml)-coated plates. After 1 week of differentiation, 100 nM Cytosine-β-d-arabinofuranoside hydrochloride (Sigma) was added to reduce proliferation of remaining neural progenitors.^84^ A similar strategy was used for imaging plates (MaTek Lifesciences). Differentiations were analysed 30–40 days post-plating. For later passage of neural progenitors, we used a CD271^−^/CD133^+^/CD184^+^ (BioLegend) flow-cytometry purification strategy to remove minority neural crest contaminants (CD271^+^) that can expand over time, as has been done previously.^85^

#### Western blot analyses of reprogrammed cortical neurons

Cell lysates from wild-type and mutant human cortical neurons at 30–40 days of differentiation were lysed in Laemmli sample buffer and boiled at 96°C for 5 min. Cell lysates were sonicated by probe sonication to shear cellular debris and genomic DNA. Proteins were separated by 10% Bis-Tris gel (Invitrogen) and transferred to nitrocellulose membrane. After blocking in 5% milk/TBS or BSA/TBS, membranes were incubated with primary antibodies (anti-total tau (tau 46) (sc-32274) from Santa Cruz; anti-tau AT8 (MN1020) and anti-GAPDH (MA5-15738 and PA5-85074) from Invitrogen; anti-TTL (13618-1-AP) from Proteintech; anti-detyrosinated tubulin (MAB5566) from Sigma-Aldrich; anti-Δ2 antibody (AB3203) from Millipore) at 4°C overnight and 1 h with appropriate secondary antibodies (LI-COR Biosciences). Image acquisition was performed with an Odyssey infrared imaging system (LI-COR Biosciences) and analysed with Odyssey software.

### Statistical analysis

Data analyses, statistical comparisons and graphs were generated using GraphPad prism or the R programming language. Statistical analysis of differences between two groups was performed using Student’s *t-*tests for populations with Gaussian distribution or else with Mann–Whitney’s test. When comparing three or more univariate samples we used one-way ANOVA, except for Fig. 2F and Supplementary Figs 4 and 6, in which we used the non-parametric Kruskal–Wallis test due to non-normality of the samples. When ANOVA indicated that the factor under study had a significant effect, *post hoc* comparisons between factor levels (using the unexplained variance calculated in the ANOVA) were performed with the Dunnett or Sidak tests, depending on whether comparisons were, respectively, with the sole control condition or between any two conditions. *Post hoc* comparisons following Kruskal–Wallis test were done with the non-parametric Dunn test. For bivariate statistics we used two-way ANOVA, with type II sum of squares when samples were unbalanced (to avoid confusion between factors). *Post hoc* comparisons were performed between non-weighted marginal means, using Dunnett or Sidak tests, depending on whether all-versus-control or all-versus-all comparisons were needed. The calculations were performed with the R car and emmeans packages. For Fig. 3B–F, as regular two-way ANOVA was not suitable we used a linear mixed model and calculation of model coefficients by restricted maximum likelihood estimation (using the R lmer package). The significance of fixed effects (Braak stage and brain region) was then evaluated by Wald type II F tests (with Kenward–Roger correction) of the null hypothesis for each of the model coefficients. *Post hoc* comparisons were run by Sidak tests. In Fig. 3H, to determine whether the distributions of immunoreactivity values in control and Alzheimer’s disease neuronal populations were significantly different we used the Kolmogorov–Smirnov test. In Fig. 5E, we used an overall chi-square test that showed that the proportion of pruned spines significantly depended on at least one of the two factors under study (oAβ treatment and microtubule invasion). Then to evaluate the specific association of spine resistance with microtubule entry, we calculated the odds ratio of spine pruning in vehicle versus oAβ-treated neurons, separately for microtubule-invaded and non-invaded spines. The significance of the difference between the two odds ratios was assessed with the Woolf-test of homogeneity of odds ratios, using the R vcd package. In Supplementary Fig. 5B and C an overall chi-square test was used on two factors under study (oAβ treatment and microtubule invasion) and the four possible spines morphological fates. Mean differences were considered significant at *P* < 0.05 (*P < 0.05; **P < 0.01; ***P < 0.001 and ****P < 0.0001). Some exact *P*-values are indicated in text or figures.

### Data availability

The datasets generated and/or analysed during the current study are available from the corresponding authors on request.

## Results

### Inhibition of tubulin retyrosination induces age-dependent synaptic defects

**Figure 1:**
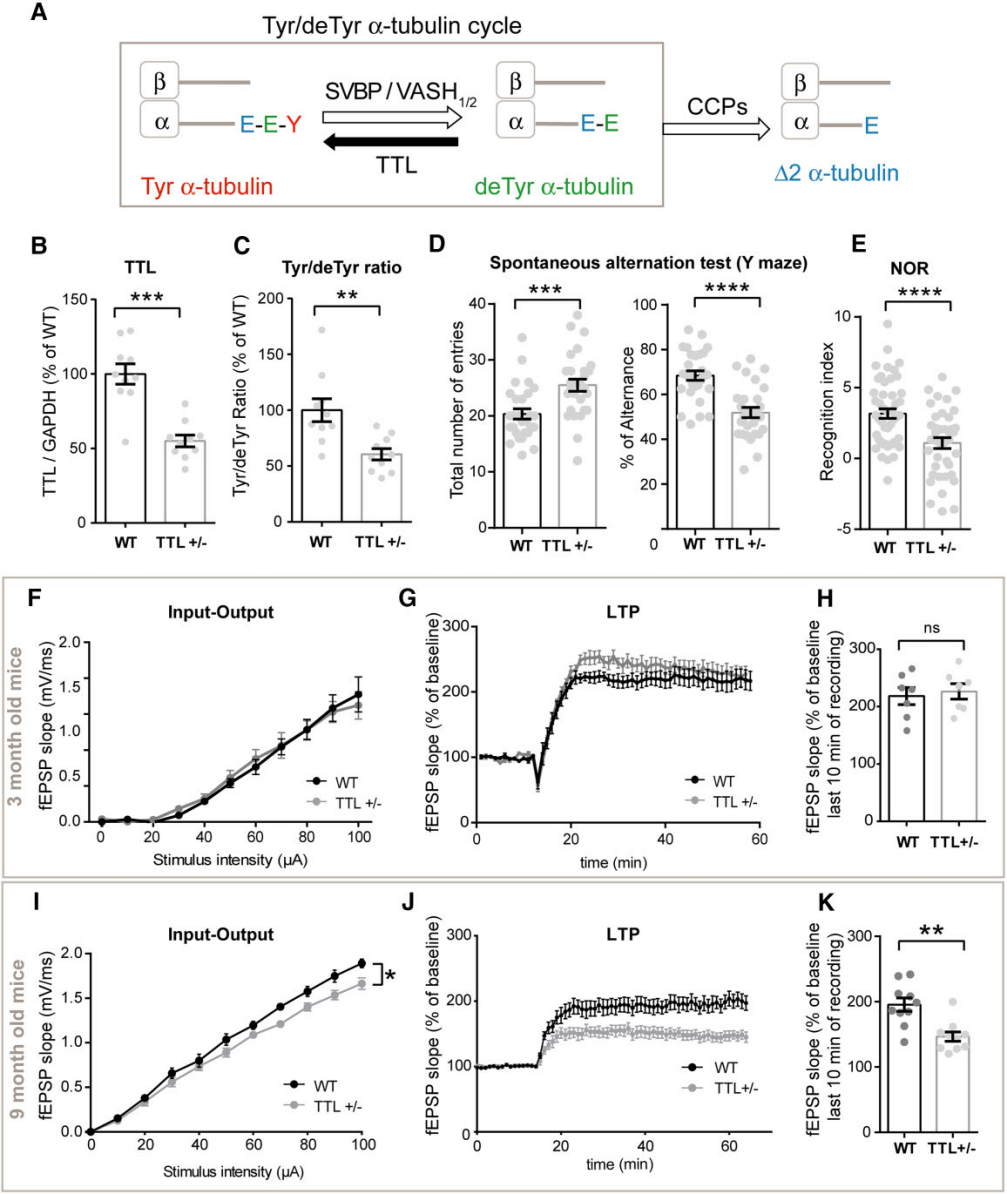
**TTL reduction induces early memory defects and age-dependent alteration of synaptic plasticity.** (**A**) Schematic representation of α-tubulin tyrosination/detyrosination cycle. CCPs = cytosolic carboxypeptidases. (**B** and **C**) Relative amount of TTL (normalized with GAPDH) and tyrosinated/detyrosinated tubulin ratio, in protein extracts from hippocampi of 3-month-old wild-type (WT) and *TTL^+/−^* mice. Graphs represents mean ± SEM. Mann–Whitney test, **P < 0.01, ****P < 0.0001. *n* = 10 independent experiments for each genotype. (**D**) Spontaneous alternation in Y-maze test. Total number of arm entries and percentage of alternance of 3-month-old WT and *TTL^+/−^* mice. Graph represents mean ± SEM. *n* = 28 for WT and *TTL^+/−^* mice. Student’s *t*-test, ***P < 0.001, ****P < 0.0001. (**E**) Novel Object Recognition test. Recognition index (time spent exploring the novel object minus the time spent exploring the two familiar objects, in seconds) of 3-month-old WT and *TTL^+/−^* mice, measured 1 h after familiarization. Mean ± SEM, *n* = 48 and 40 for WT and *TTL^+/−^* mice, respectively. Student’s *t*-test, ****P < 0.0001. (**F**) Input/output (I/O) curves of 3-month-old WT and *TTL^+/−^* mice slices. Curves were constructed by plotting mean fEPSPs slopes ± SEM as a function of stimulation intensity. Two-way ANOVA, Genotype × Stimulation intensity interaction is not significant [*F*(10,80) = 0,3845, *P* = 0.9500]. *n* = 5 slices from three WT mice and *n* = 5 slices from three *TTL^+/−^* mice. (**G**) LTP of 3-month-old WT and *TTL^+/−^* mice. Curves represent normalized mean of fEPSPs slopes ± SEM as a function of time before and after LTP induction. (**H**) Graph showing normalized mean of fEPSPs slopes ± SEM for the last 10 min of recording in WT and *TTL^+/−^* mice. Mann–Whitney test, ns = not significant (*P* = 0.8048). *n* = 7 slices from three WT mice and *n* = 7 slices from three *TTL^+/−^* mice. (**I**) Input/output (I/O) curves of 9-month-old WT and *TTL^+/−^* mice slices. Two-way ANOVA, Genotype × Stimulation intensity interaction [*F*(10,220) = 1,923, *P = 0.0433]. *n* = 12 slices from five WT mice and *n* = 12 slices from five *TTL^+/−^* mice. (**J**) LTP of 9-month-old WT and *TTL^+/−^* mice. (**K**) Graph showing normalized mean of fEPSPs slopes ± SEM for the last 10 min of recording in WT and *TTL^+/−^* mice. Mann–Whitney test, **P = 0.0021; *n* = 10 slices from four WT mice and *n* = 10 slices from four *TTL^+/−^* mice.

Dynamic microtubules are crucial for synaptic plasticity and known to bear tyrosinated tubulin, and so we directly examined whether perturbation of the tubulin tyrosination/detyrosination cycle (Fig. 1A) affects synaptic function. As total genetic ablation of tubulin tyrosine ligase is perinatally lethal in mice,^60^ we used *TTL^+/−^* mice that are viable and fertile. First, we confirmed that in protein extracts from hippocampi of 3- and 9-month-old *TTL^+/−^* mice both tubulin tyrosine ligase protein levels and tyrosinated/detyrosinated tubulin ratio were significantly reduced compared to wild-type mice (*TTL^+/−^* = −44.95 ± 3.95% and −48.94 + 2.61% of wild-type and tyrosinated/detyrosinated tubulin ratio = −39.46 ± 5.04% and −37.08 ± 4.71% of wild-type for 3- and 9-month-old mice, respectively, Fig. 1B and C and Supplementary Fig. 1A–C).

We performed spontaneous alternation in Y-maze and novel object recognition memory tests (Fig. 1D and E). These memory tests were selected because they broadly assess function of cognitive domains that correlate with neural circuitry disrupted early in Alzheimer’s disease, including the hippocampus,^86^ and have been useful to reveal memory defects in preclinical models of β-amyloidosis and tauopathy.^87,88^*TTL^+/−^* mice exhibited robust deficits in spontaneous alternation in Y-maze (20.36 ± 0.91 versus 25.50 ± 1.09 number of entries and 68.44 ± 2.13 versus 51.88 ± 2.29% of alternation for wild-type and *TTL^+/−^* mice, respectively) (Fig. 1D). Also, in the novel object recognition test, *TTL^+/−^* mice spent significantly less time exploring the novel object than wild-type mice (delta between new and familiar object of 3.17 ± 0.33 versus 1.08 ± 0.37 sec for wild-type and *TTL^+/−^* mice, respectively) (Fig. 1E). *TTL^+/−^* mice showed no defect in locomotor activities and sensorimotor functions as well as intact hippocampus-dependent spatial memory when assessed by the Morris Water Maze Test, consistently with lack of manifested spatial navigation defects in most preclinical Alzheimer’s disease models at a young age^89^ (Supplementary Fig. 2). These data demonstrate that reduced tyrosinated/detyrosinated tubulin ratio impairs spatial working and short-term recognition memory with negligible effects on sensorimotor circuit development, hyperactivity and spatial navigation, a behavioural profile that is compatible with the cognitive decline observed in preclinical models of Alzheimer’s disease.^86–89^

Next, we investigated hippocampal synaptic transmission in 3- and 9-month-old wild-type and TTL heterozygous mice. The efficacy of basal excitatory synaptic transmission was determined by field recordings of postsynaptic excitatory responses elicited by a range of electrical stimuli of axonal CA3-CA1 Schaffer collateral fibres, in hippocampal slices. While in 3-month-old animals, the input/output (I/O) curves revealed no differences between genotypes, in 9-month-old mice, we observed a significantly weaker postsynaptic response in *TTL^+/−^* than in wild-type animals (Fig. 1F and I) indicating defective basal synaptic transmission in older *TTL^+/−^* mice. Furthermore, application of a theta-burst LTP protocol showed no difference in potentiation in 3-month-old mice between wild-type and *TTL^+/−^* mice (Fig. 1G and H), but a reduced potentiation in 9-month-old *TTL^+/−^* compared to wild-type mice (Fig. 1J and K, −25.04 ± 3.68% of wild-type).

Altogether, these data demonstrate that a reduction in TTL expression results in loss of tyrosinated tubulin *in vivo*, early memory defects and age-dependent hippocampal synaptic dysfunction that affects both basal transmission and activity-dependent plasticity.

### Inhibition of tubulin retyrosination affects dendritic spine density

**Figure 2:**
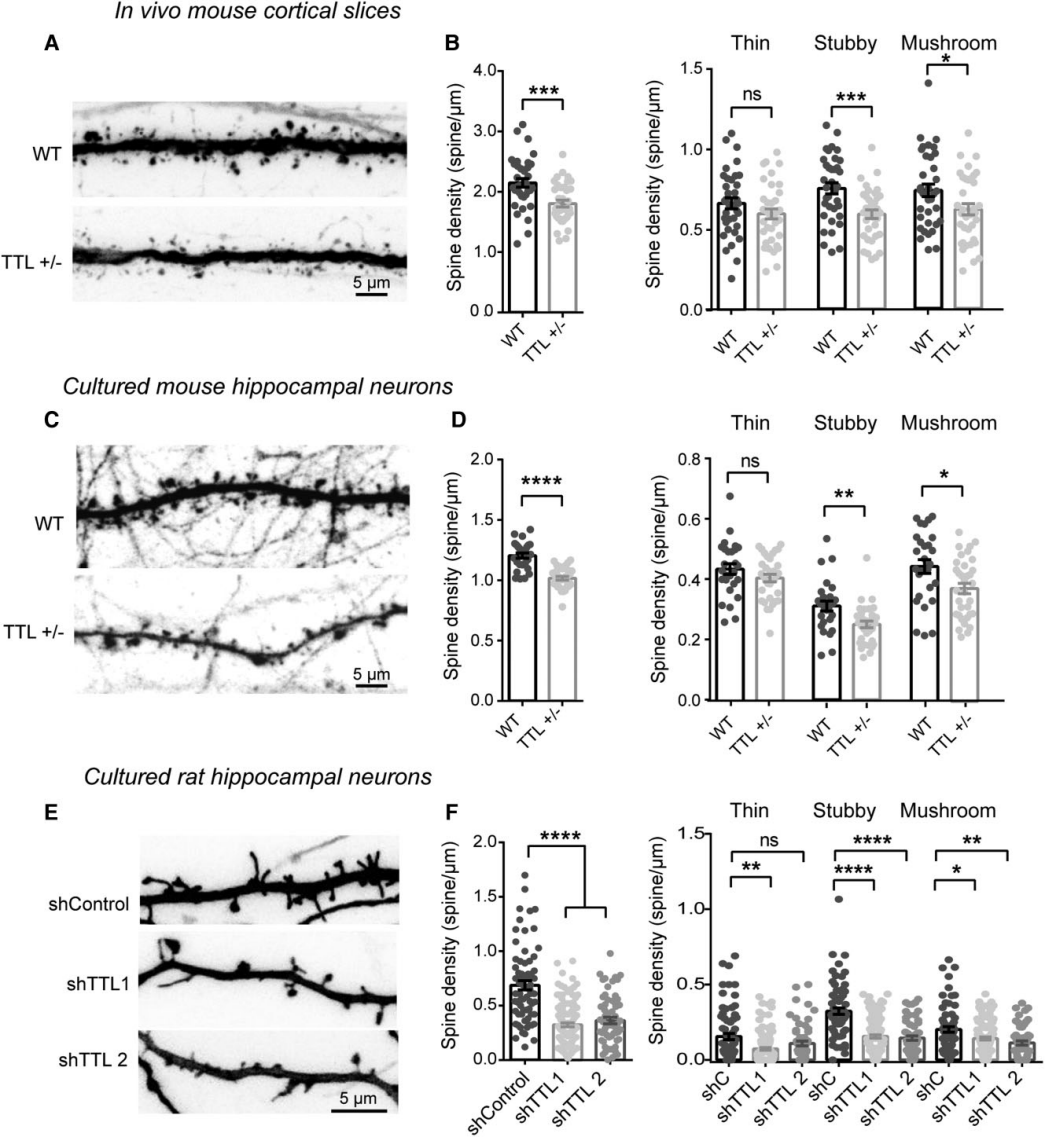
**TTL reduction decreases dendritic spine density *in vivo* and in cultured neurons.** (**A**) Confocal images showing representative examples of dendritic segments of cortical neurons from 4-month-old Thy1-eYFP-H wild-type (WT) and Thy1-eYFP-H *TTL^+/−^* mice. (**B**) Total dendritic spine density, or that of each different morphological type of spines, is represented for Thy1-eYFP-H WT and Thy1-eYFP-H *TTL^+/−^* cortical neurons. Graphs represent mean ± SEM. *n* = 36 neurons from four independent animals of each genotype. Student’s *t*-test, *P < 0.05; ***P < 0.001 and ns = not significant. (**C**) Confocal images showing representative examples of the dendritic segments of GFP-expressing WT and *TTL^+/−^* hippocampal neurons in culture at 17 DIV. (**D**) Total dendritic spine density, or that of each different morphological type of spines are represented for WT and *TTL^+/−^* hippocampal cultured neurons. Graphs represent mean ± SEM. *n* = 27 and *n* = 34 neurons from WT and *TTL^+/−^* embryos from at least three independent cultures. Student’s *t*-test, *P < 0.05; **P < 0.01; ****P < 0.0001 and ns = not significant. (**E**) Confocal images showing representative examples of dendritic segments of DiOilistic labelled WT rat hippocampal neurons in culture at 21 DIV, infected with control shRNA or shRNA targeting tubulin tyrosine ligase (shTTL1 and shTTL2). (**F**) Total dendritic spine density or that of each different morphological type of spines, of hippocampal neurons infected with control shRNA (non-coding shRNA) or two independent shRNA lentiviruses targeting tubulin tyrosine ligase (sh*TTL1* and sh*TTL2*). Graphs represent mean ± SEM. *n* = 71, *n* = 124 and *n* = 60 neurons from control shRNA, sh*TTL1* and sh*TTL2*, respectively, from at least three independent cultures. Kruskal–Wallis with Dunn’s multi-comparison test, *P < 0.05; **P < 0.01; ****P < 0.0001; ns = not significant. Spine assignation to thin, stubby or mushroom categories was performed according to morphological parameters described in Supplementary Fig. 5.

We examined the effects of TTL reduction at the level of individual neurons by measuring dendritic spine density and morphology both *in vivo* and using neurons in primary neuronal culture. Dendritic spines are often classified in three morphological types, corresponding to successive developmental stages: thin, stubby and mushroom-like spines.^90^ For *in vivo* evaluation, *TTL^+/^^−^* mice were crossed with Thy1-e-YFP-H transgenic mice to visualize dendritic spines, and spine density evaluated in layer V cortical neurons.^74^ These neurons express moderate YFP levels, allowing accurate quantification of spine density (Fig. 2A), in contrast to hippocampal neurons in which expression levels were too high for proper assessment. Confocal microscopy of *in situ* cortical neurons from *TTL^+/−^*-Thy1-eYFP-H mice showed a 15.97 ± 2.6% decrease in dendritic spine density compared to WT^+/−^-Thy1-eYFP-H littermates (2.147 ± 0.07 and 1.804 ± 0.05 spines/µm for wild-type and *TTL^+/−^*, respectively). The decrease mainly affected mature forms of dendritic spines (Fig. 2A and B). A comparable drop in mature spines (−15.53 ± 1.2% of wild-type) was observed in cultured hippocampal neurons obtained from *TTL^+/−^* embryos (1.204 ± 0.021 and 1.017 ± 0.014 spines/µm for wild-type and *TTL^+/−^*, respectively, Fig. 2C and D). Similar results were obtained when acute TTL knock-down was performed in rat hippocampal neurons using two independent tubulin tyrosine ligase-targeting shRNAs (Fig. 2E and Supplementary Fig. 1D and F). Tubulin tyrosine ligase silencing resulted in an accumulation of Δ2 tubulin (Supplementary Fig. 1E and G) and induced a dramatic reduction of dendritic spine density (Fig. 2E and F, −52.88 ± 2.67%; −47.14 ± 4.30% of wild-type for shRNA1, shRNA2 treated neurons, respectively) with values similar to those observed in TTL knock-out neurons (Supplementary Fig. 3, −41.17 ± 1.25% of wild-type for TTL knock-out neurons).

Together, these results show that reducing TTL expression affects the density of dendritic spines *in vitro* and *in vivo*, providing evidence for a novel role for tubulin retyrosination in regulating structural plasticity.

### Tubulin retyrosination is perturbed in Alzheimer’s disease

**Figure 3:**
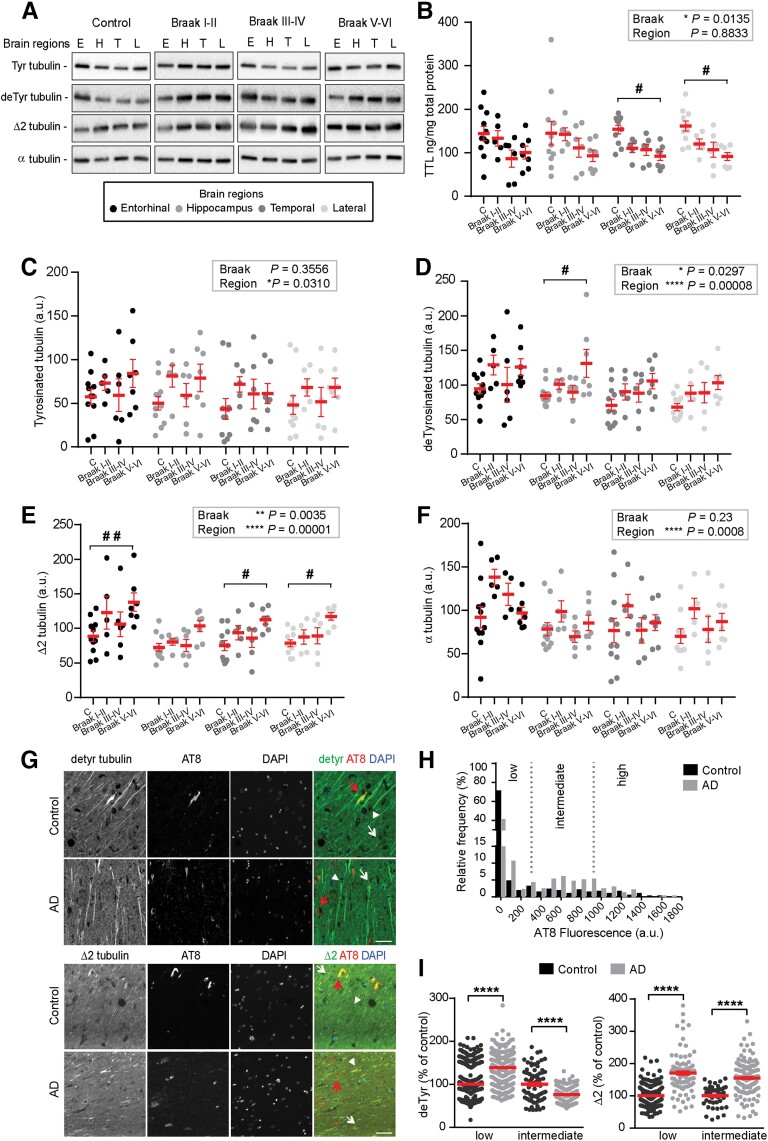
**Loss of tubulin tyrosine ligase and increased non-tyrosinated tubulin levels in sporadic Alzheimer’s disease brain samples.** (**A**) Representative immunoblot analysis of tyrosinated, detyrosinated, Δ2 and α tubulin levels in brain homogenates from entorhinal cortex (E), hippocampus (H), temporal (T) and lateral prefrontal cortex (L) from control, early Alzheimer’s disease (Braak I–II), middle Alzheimer’s disease (Braak III–IV) and late Alzheimer’s disease (Braak V–VI) patients. In each blot an internal standard corresponding to a wild-type (WT) sample was used for normalization and considered as 100% and the values for each unknown sample were calculated as a percentage of this standard (see ‘Material and methods’ section). (**B**–**F**) Quantification of tubulin tyrosine ligase (TTL) protein expression, modified tubulins (tyrosinated, detyrosinated and Δ2 tubulin) and α tubulin levels in each brain region from control and Alzheimer’s disease patients. Graphs represent mean ± SEM. The dependence of protein levels on, respectively, clinical stage and brain area was quantitated in each case using a linear mixed model, with Braak stage and brain region as fixed effect factors. Boxed *P*-values measure the overall significance of these factors (type II Wald F test of model coefficients). In each brain area, *post hoc* testing of variations due to individual Braak stages was performed by Dunnett’s test of differences with control. Significance levels are indicated as follows: ^#^*P* < 0.05 and ^##^*P* < 0.01. *n* = 11, *n* = 5, *n* = 6, and *n* = 7 for Control, Braak I–II, Braak III–IV and Braak V–VI Alzheimer’s disease patient brains, respectively. Each sample was analysed in triplicate. (**G**) Representative images of detyrosinated, Δ2 tubulin and phospho-tau in pyramidal neurons of hippocampi from control and Alzheimer’s disease patients. Dual immunostaining of detyrosinated (*upper panel*) or Δ2 tubulin (*lower panel*) and AT8-reactive phospho-tau, combined with nuclear staining with DAPI, was performed on sections of control and Alzheimer’s disease patient hippocampi. Neurons with low (white arrowheads), intermediate (white arrows) or high (red arrows) levels of AT8 immunofluorescence are shown. Scale bar = 50 µm. (**H**) Relative frequency distribution of phospho-tau (AT8) immunofluorescence levels (arbitrary units) in pyramidal neurons of control and Alzheimer’s disease brains. Low, intermediate and high phospho-tau groups were defined based on fluorescence intensity. Two-sample Kolmogorov–Smirnov test, ****P < 0.0001. (**I**) Intensity of detyrosinated tubulin (*left* graph) or Δ2 tubulin (*right* graph) immunofluorescence in pyramidal cell bodies of Alzheimer’s disease hippocampal neurons relative to control, shown as a function of AT8 labelling level. Data are presented as mean ± SEM. For detyrosinated tubulin, *n* = 382 and *n* = 67 neurons in controls and *n* = 296 and *n* = 162 for Alzheimer’s disease neurons in low and intermediate phospho-tau groups, respectively. For Δ2 tubulin, *n* = 249 and *n* = 45 neurons in controls and *n* = 91 and *n* = 133 for Alzheimer’s disease neurons in low and intermediate phospho-tau groups, respectively. Mann–Whitney test, ****P < 0.0001.

The synaptic and behavioural defects observed when levels of tyrosinated tubulin are perturbed raised the question as to whether dysregulation of tubulin retyrosination is a feature of Alzheimer’s disease, a neurodegenerative disorder in which synaptic pathology is prominent at early stages. We performed a detailed analysis of the relative amount of TTL, tyrosinated, detyrosinated and Δ2 tubulins in post-mortem human brain tissues from sporadic Alzheimer’s disease patients and age-matched controls using enzyme-linked immunosorbent assay (ELISA) and immunoblots. For these analyses, each Alzheimer’s disease brain was histologically analysed according to Braak’s criteria^91^ to discriminate early (Braak I–II), middle (Braak III–IV) and late Alzheimer’s disease stages (Braak V–VI), as shown in Supplementary Table 1. Alzheimer’s disease sequentially affects the entorhinal cortex (E), hippocampus (H), temporal cortex (T) and lateral prefrontal cortex (L). We analysed TTL levels and the different α-tubulin forms in protein extracts prepared from these four brain regions of Alzheimer’s disease patients and controls (Fig. 3A–F). Global analysis indicated a statistically significant effect of Braak stages on TTL content [*F*(3,25) = 4.3454, *P = 0.0135, Fig. 3B, grey box]. *Post hoc* comparison of TTL content in control and Alzheimer’s disease brains showed a significant decrease in temporal and lateral prefrontal cortex of Alzheimer’s disease patients (^#^*P* = 0.0322 and ^#^*P* = 0.012, respectively for Braak V–VI versus controls, Fig. 3B). No significant effect of brain region on TTL content was observed [*F*(3,75) = 0.2185, *P* = 0.8833, Fig. 3B, grey box] suggesting that the TTL decrease observed in Alzheimer’s disease samples affects the whole brain. Regarding tyrosinated tubulin levels, a global analysis indicated that there was no significant dependence on Braak stage [*F*(3,25) = 1.1336, *P* = 0.3556, Fig. 3C, grey box]. For detyrosinated and Δ2 tubulin levels, the Braak stage had a global significant effect [*F*(3,25) = 3.515, *P = 0.0297 and *F*(3,25) = 5.877, **P = 0.0035 for detyrosinated and Δ2 tubulins, respectively, Fig. 3D and E, grey boxes]. *Post hoc* comparisons in each brain region as a function of Braak stage, indicated that the detyrosinated tubulin content significantly accumulated in the hippocampus of patients with advanced disease (Fig. 3D, ^#^*P* = 0.0131 for Braak V–VI versus controls). Further, the amount of Δ2 tubulin increased in all regions in Alzheimer’s disease samples, as compared to controls (Fig. 3E, ^##^*P* = 0.0018, *P* = 0.0584, ^#^*P* = 0.0195 and ^#^*P* = 0.0144 for entorhinal, hippocampus, temporal and lateral cortex, respectively, for Braak V-VI versus controls). Importantly, the amount of total tubulin did not vary with disease stage [*F*(3,25) = 1.54, *P* = 0.23, Fig. 3F], confirming that the increase observed in disease samples was selective for detyrosinated and Δ2 tubulins. To note, the levels of tyrosinated, detyrosinated and Δ2 tubulins, as well as total tubulin, were significantly different among brain regions [Fig. 3C–F grey boxes; *F*(3,75) = 3.1183, *P = 0.0310; *F*(3,75) = 8.190, ****P = 0.00008; *F*(3,75) = 10.091, ****P = 0.00001; *F*(3,75) = 6.19, ****P = 0.0008 for tyrosinated, detyrosinated, Δ2 and total tubulin, respectively], a feature mostly attributable to larger concentration of tubulin in the entorhinal cortex extracts than in the other brain region samples.

Altogether, these results indicate that in Alzheimer’s disease, a global TTL impairment is present from an early stage of the neurodegeneration process and is associated with increased amounts of non-tyrosinated tubulin.

We next analysed modifications in non-tyrosinated tubulin content *in situ* by performing an immunocytochemistry study of Alzheimer’s disease brains. We performed a semiquantitative immunofluorescence analysis of cell bodies and proximal dendrites of randomly selected individual pyramidal cells in the anterior hippocampal formation of sections from Alzheimer’s disease and control tissue (Fig. 3G–I and Supplementary Table 2). Each selected neuron was classified for tau pathology with either low, intermediate or high level of AT8 labelling (Ser202 and Thr205 phospho-tau antibody) and the mean intensity of detyrosinated and Δ2 tubulin staining was calculated. As expected, strongly AT8-reactive neurons were far more frequent in the Alzheimer’s disease samples, consistent with the pathological scoring of control and Alzheimer’s disease post-mortem human brains (Fig. 3G and H and Supplementary Table 2). Interestingly, we found that Alzheimer’s disease neurons with relatively low levels of phospho-tau, and thus presumably at an early stage of the degeneration process, were significantly enriched in detyrosinated and Δ2 tubulins compared to non-diseased neurons (Fig. 3I, ****P < 0.0001 for each). In contrast, Alzheimer’s disease neurons with intermediate AT8 staining still displayed significant enrichment in Δ2 tubulin compared to non-diseased neurons (Fig. 3I, ****P < 0.0001) but a lower level of detyrosinated tubulin, presumably as a result of advanced neurodegeneration and/or accelerated conversion of detyrosinated to Δ2 tubulin in diseased brains. These *in situ* results confirmed the accumulation of non-tyrosinated tubulin in pyramidal neurons in Alzheimer’s disease and indicated that it may occur at an early stage of the neurodegeneration process.

**Figure 4:**
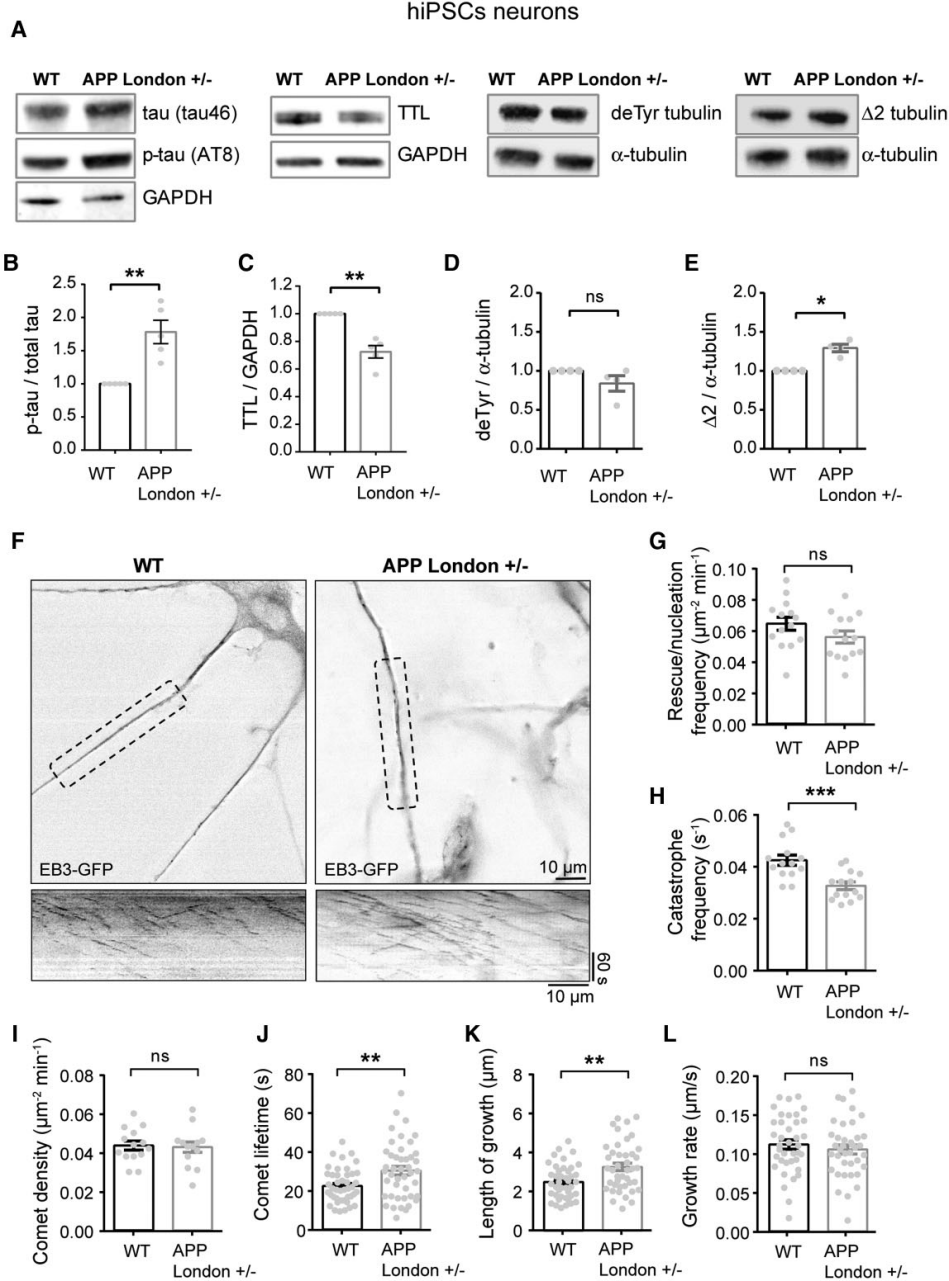
**Loss of TTL and increased non-tyrosinated tubulin levels correlate with inhibition of microtubule dynamics in human cortical APP-London neurons.** (**A**) Immunoblot analysis of phospho-specific tau (AT8), total tau (tau46), TTL, detyrosinated tubulin and Δ2 tubulin from lysates of human cortical neurons, derived from wild-type (WT) and APP-London (V717I) knocked-in iPSCs isogenic lines. GAPDH was used for tau and TTL normalization and total tubulin for modified tubulins. Immunoblot quantifications of phospho-tau normalized to total tau (**B**), TTL (**C**), detyrosinated (**D**) and Δ2 tubulin (**E**). Data are expressed as a ratio of WT and graphs represent mean ± SEM. *n* = 5, *n* = 5, *n* = 4 and *n* = 4 independent neuronal differentiation experiments for **B**, **C**, **D** and **E**, respectively. Mann–Whitney test, ns = not significant, *P < 0.05, **P < 0.01. (**F**) WT and APP-London human cortical neurons expressing EB3-GFP. Representative neurites (dashed boxes) from human cortical neurons were analysed for microtubule dynamics and kymographs of these regions are shown below. Scale bar: 10 μm. (**G**–**L**) Parameters of microtubule dynamics are represented as mean ± SEM. *n* = 14 neurites from WT and APP-London neurons for **G** to **I**, and *n* = 44 comets for **J**, *n* = 42 comets for **K** and *n* = 38 comets for **L**, from WT and APP-London neurons, respectively. Student’s *t*-test, ns = not significant, **P < 0.01 and ***P < 0.001.

To explore whether perturbation of tubulin retyrosination and microtubule dynamics was a hallmark of familial Alzheimer’s disease, we used isogenic human iPSC lines in which the Alzheimer’s disease-linked London mutation (V717I) was knocked-in via CRISPR–Cas9 into one allele of the *APP* gene to replicate the genuine familial Alzheimer’s disease genotype.^83^ Human iPSCs harbouring the London mutation and the isogenic control parent line were differentiated *in vitro* into human cortical neurons via a neural progenitor intermediate as previously described.^83,84^ After 30–40 days of differentiation, a time at which differentiated cortical neurons establish synapses, neurons were lysed and TTL, detyrosinated and Δ2 tubulin levels analysed by immunoblotting. At this stage of differentiation, the mutant neurons accumulated tau protein, which was hyperphosphorylated (tau46 and AT8, Fig. 4A and B), confirming the occurrence of a previously described pathological feature associated with this APP mutation.^68^ Consistent with our observations of brain samples, neurons with mutant APP displayed a significant reduction in TTL content (Fig. 4C), an increase in Δ2 tubulin levels, and showed a trend in the accumulation of detyrosinated tubulin compared to isogenic controls (Fig. 4D and E).

We next directly examined microtubule dynamics in human neurons by transiently expressing the microtubule plus-end binding protein, EB3-eGFP to track the dynamic behaviour of microtubule plus ends (Fig. 4F–L). We found that in neurons with mutant APP, while comet density, growth rate and rescue/nucleation frequency were unchanged (Fig. 4G, I and L), catastrophe frequency (Fig. 4H) was significantly reduced compared to wild-type controls with a corresponding increase in comet lifetime and length of growth (Fig. 4J and K). These observations are consistent with mutant APP-dependent inhibition of microtubule dynamics by inducing resistance to undergo microtubule depolymerization.

Together, our results indicate that tubulin retyrosination is affected in sporadic and familial Alzheimer’s disease and that inhibition of microtubule dynamics observed in mutant APP human neurons is consistent with a disrupted tubulin tyrosination/detyrosination cycle.

### Tubulin retyrosination protects neurons from oAβ synaptotoxicity and promotes microtubule invasion into spines

**Figure 5:**
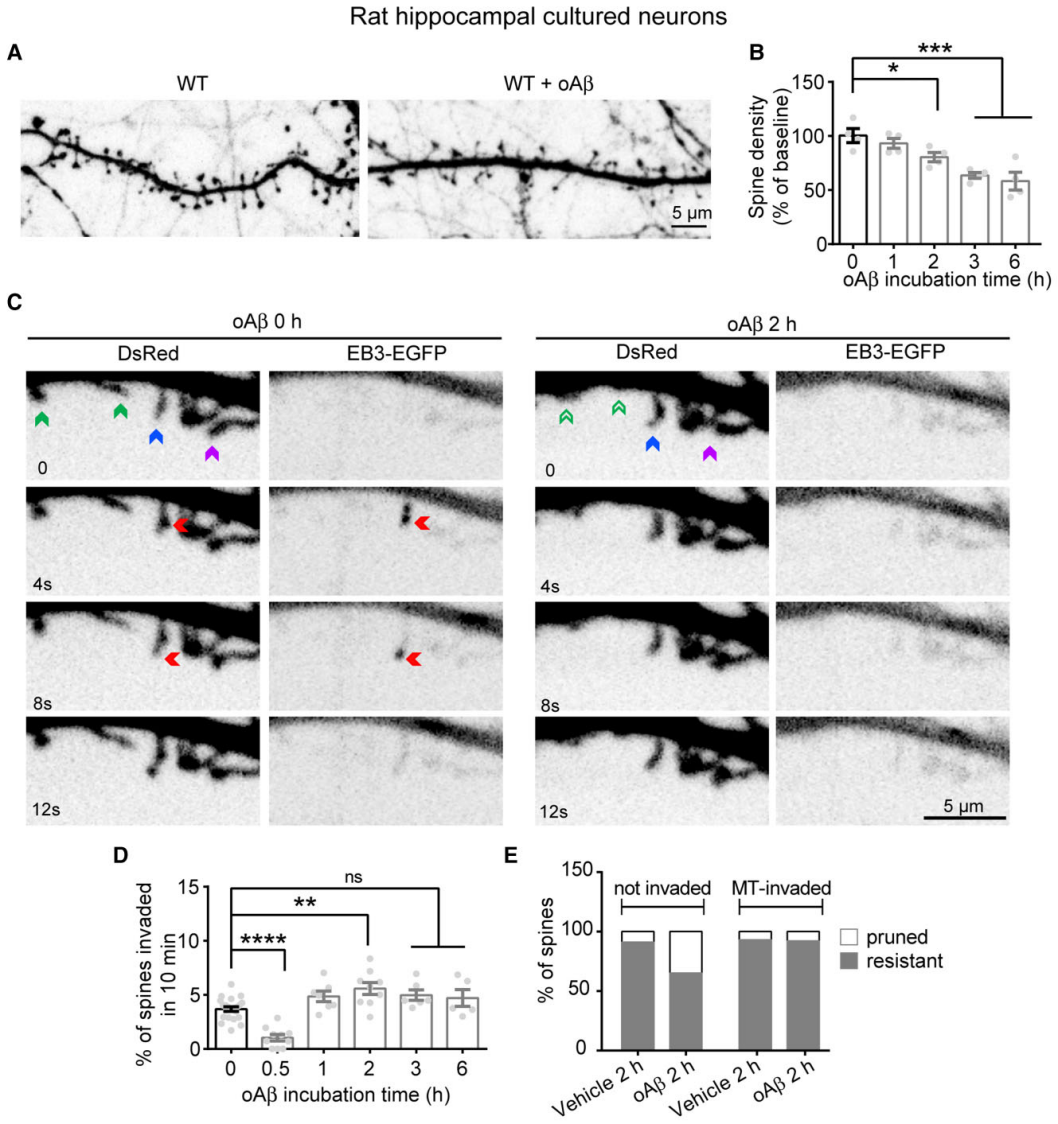
**Acute oAβ treatment affects spine invasion by dynamic microtubules in neurons.** (**A**) Confocal images showing representative examples of dendritic segments of eGFP expressing wild-type (WT) rat hippocampal neurons (17 DIV) treated with DMSO or with 250 nM of oAβ for 2 days. (**B**) Graphs of the percentage of dendritic spine density in WT cultured neurons incubated with oAβ over 6 h. Data are expressed as a percentage of baseline and graphs represent mean ± SEM. *n* = 4 neurons analysed over time. One-way ANOVA with Dunnett’s multiple comparison test, *P < 0.05 and ***P < 0.001. (**C**) Representative stills from videos of a WT neuron (21 DIV) transfected with DsRed and EB3-eGFP to visualize dendritic spines and the growing plus ends of microtubules, before and 2 h after oAβ treatment. Spines that will prune are highlighted with a green arrow at time 0, and with an empty green arrow after 2 h of oAβ treatment. The spine that will be invaded by a microtubule is highlighted with a blue arrow at time 0 and persists after 2 h of oAβ treatment. Microtubule invasion into the spine is highlighted with a red arrow. Spines that are not invaded but persist after oAβ treatment are highlighted with arrows in magenta. (**D**) Percentage of spines invaded by microtubules before and after oAβ exposure at the indicated times. Graphs represent mean ± SEM. *n* = 22, *n* = 10, *n* = 9, *n* = 6 and *n* = 5 neurons at each time point. One-way ANOVA with Dunnett’s multiple comparison test, ns = not significant, **P < 0.01 and ****P < 0.0001. Number of spines: 402, 150, 411, 191, 321, 342 and 285 for control and amyloid-β (0.5 h, 1 h, 1.5 h, 2 h, 3 h and 6 h) conditions, respectively. (**E**) Total percentage of spine pruning or resistance to vehicle or oAβ incubation. Graph represents the mean percentage of non-invaded spines (*left*) or microtubule-invaded spine fate (*right*) for either fate. Spines invaded by microtubules (*n* = 45 and *n* = 24) and spines non-invaded by microtubules (*n* = 43 and *n* = 43) for vehicle and oAβ conditions, respectively. Microtubule-invaded spines were significantly more resistant to oAβ-induced pruning than non-invaded spines (overall dependence of the spine pruning rate on microtubule invasions and oAβ treatment: *X*^2^ = 43.64, 4 df, ****P < 0.0001, chi-square test; odds ratio of resistance to oAβ in microtubule-invaded versus; non-invaded spines (1.15 versus 5.44, *X*^2^ = 5.27, 1 df, *P = 0.021, Woolf test).

APP variants such as the London mutant generate larger amounts of amyloid-β peptide (1–42)^92^ and soluble oAβ has been proposed to contribute to loss of synapses at an early stage of neurodegeneration in Alzheimer’s disease.^93^ We analysed the consequences of oAβ on the behaviour of spine-invading microtubules in cultured hippocampal neurons. First, we observed that neurons exposed to oAβ lost their spines in a time-dependent manner (−6.80 ± 4.63, −19.56 ± 4.41; −36.42 ± 2.79 and −40.33 ± 6.57% of control cells after 1, 2, 3 and 6 h of oAβ exposure, respectively) (Fig. 5A and B). Next, we analysed the dynamics of microtubule invading into individual spines of neurons cotransfected with plasmids expressing EB3-eGFP and DsRed as a cell filler, in response to oAβ (Fig. 5C). The dynamic parameters of spine-invading microtubules (length of growth, comet lifetime, growth rate) and spine invasion lifetime were not affected by oAβ (Supplementary Fig. 4). However, oAβ acutely inhibited microtubule entry into spines at 0.5 h, while inducing a time-dependent renormalization of the fraction of microtubule-invaded spines starting at 2 h (3.68 ± 0.21, 1.03 ± 0.29, 5.58 ± 0.54, 4.97 ± 0.48 and 4.70 ± 0.77% of spines for 0, 0.5, 2, 3 and 6 h of treatment, respectively, Fig. 5D), an effect possibly due to the reduction of the total number of spines over time (Fig. 5B). We tracked and quantified the morphology of the same spines invaded or not invaded by microtubules in neurons treated with vehicle or oAβ for 2 h (Fig. 5E). In the absence of oAβ, microtubule-invaded thin spines appeared to switch more frequently to the larger stubby and mushroom spine types (Supplementary Fig. 5), a phenotype in agreement with previous observations reporting modifications of spine morphology on microtubule entry.^11^ However, in the presence of oAβ, spines that were not invaded by dynamic microtubules had a higher chance of being pruned (Fig. 5E and Supplementary Fig. 5B and C) and the non-invaded mushroom spines that did not collapse showed increased transitions to stubby or thin spines, presumably causing additional loss of synaptic strength (Supplementary Fig. 5B and C). For example, after 2 h of oAβ treatment, only 9% of microtubule-invaded spines were pruned compared to 35% of non-targeted spines (Fig. 5E).

These results indicate that oAβ causes early inhibition of microtubule entry into spines, and that these changes may be functionally related to the onset of spine pruning. The renormalization of the percentage of microtubule-invaded spines that we observed at later time points might thus reflect a relative accumulation of a class of spines that are intrinsically resistant to pruning. These results further suggest that entry of dynamic microtubules, which are mainly composed of tyrosinated tubulin, may underlie the resistance of dendritic spines to synaptic injury by oAβ.

**Figure 6:**
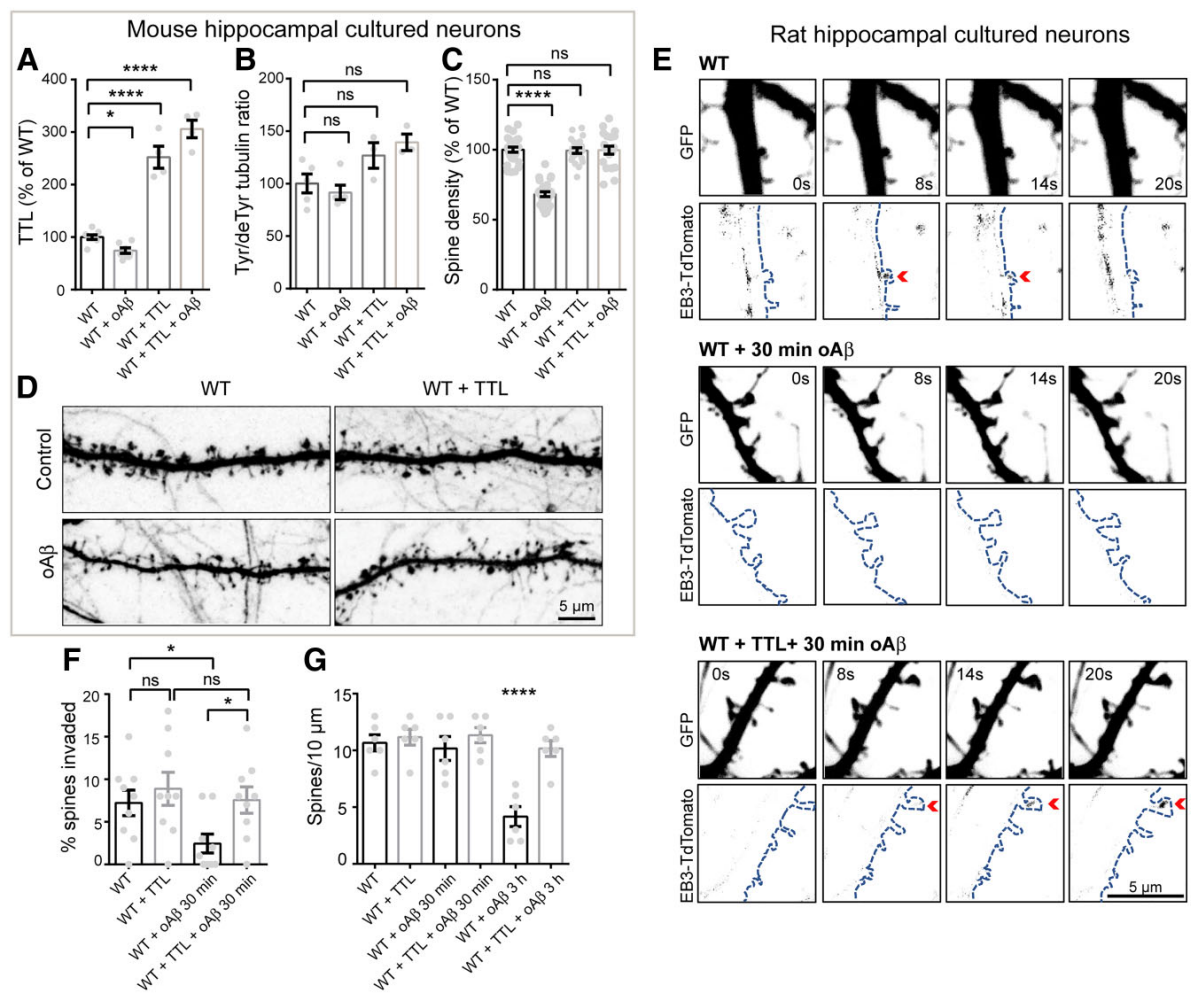
**Ectopic TTL expression rescues neurons from oAβ-induced dendritic spine loss and resumes microtubule invasions into spines.** (**A** and **B**) Immunoblot analysis of TTL (**A**) and tyrosinated/detyrosinated tubulin ratio (**B**) from wild-type (WT) mouse cortical neurons (17 DIV) transduced or not with a lentivirus expressing TTL and chronically treated with DMSO or with 100 nM oAβ. Data are expressed as a percentage of WT and graphs represent mean ± SEM. (**A**) *n* = 8, *n* = 7, *n* = 4 and *n* = 4 cultures for WT, WT+ Aβ, WT + TTL and WT + Aβ + TTL respectively. Two-way ANOVA, oAβ treatment × TTL expression interaction [*F*(1,19) = 14.6, **P = 0.0012]. All values were compared to WT, Dunnett’s multiple comparison test, *P < 0.05 and ****P < 0.0001. (**B**) *n* = 5, *n* = 5, *n* = 3 and *n* = 3 cultures for WT, WT + oAβ, WT + TTL and WT + oAβ + TTL respectively. Two-way ANOVA, oAβ treatment × TTL expression interaction [*F*(1,12) = 1.309, *P* = 0.274]. All values were compared to WT, Dunnett’s multiple comparison test, ns = not significant. (**C**) Graphs of total dendritic spine density in cultured WT neurons treated as in A. Graphs represent mean ± SEM. *n* = 27, *n* = 26, *n* = 20 and *n* = 20 neurons for WT, WT + oAβ, WT + TTL and WT + oAβ + TTL, respectively. Two-way ANOVA, oAβ treatment × TTL expression interaction [*F*(1,89) = 58.44, ****P < 0.0001]. All values were compared to WT, Dunnett’s multiple comparison test, ns = not significant and ****P < 0.0001. (**D**) Confocal images showing representative examples of dendritic segments of GFP-expressing WT hippocampal mouse neurons (17 DIV) chronically treated with DMSO or with 100 nM oAβ. (**E**) Representative stills from videos of rat WT neurons (18 to 21 DIV) transduced or not with a TTL containing lentivirus and transfected with plasmids encoding eGFP and EB3-tdTomato to visualize the dendrites and spines and the growing plus ends of microtubules, respectively. Cells were incubated with vehicle or with 250 nM of oAβ for 30 min. Microtubule invasions of spines are highlighted with a red arrow. (**F**) Percentage of spines invaded by microtubules after vehicle or oAβ exposure. Graphs represent mean ± SEM. *n* = 9 neurons for each condition. Two-way ANOVA, oAβ treatment × TTL expression interaction [*F*(1,32) = 4.76, *P* = 0.037]. Holm–Sidak’s multiple comparison test, ns = not significant, *P < 0.05. (**G**) Graphs of total dendritic spine density in cultured neurons treated as in **E** and incubated with vehicle or with oAβ for 30 min or 3 h. Graphs represent mean ± SEM. *n* = 6 neurons of each condition. Two-way ANOVA, oAβ treatment × TTL expression interaction [*F*(2,30) = 7.11, *P* = 0.003]. Holm–Sidak’s multiple comparison test, ns = not significant, ****P < 0.0001. For **F** and **G**, number of spines analysed: *n* = 119, *n* = 117, *n* = 106, *n* = 123, *n* = 75 and *n* = 106 for control, control + TTL, control + Aβ 30 min, control + TTL + Aβ 30 min, control + Aβ 3 h and control + TTL + Aβ 3 h, respectively.

We next examined the effect of chronic exposure to oAβ on TTL and tubulin tyrosination levels in primary cultured neurons. We found that 2 days of chronic 100 nM oAβ exposure resulted in a 25.77 ± 5.23% reduction in TTL content (Fig. 6A), similar to what we observed in sporadic and familial Alzheimer’s disease samples (Figs 3B and 4A and C). Acute 250 nM oAβ exposure resulted in a decline of both TTL levels and the tyrosinated/detyrosinated tubulin ratio starting at 30 min (Supplementary Fig. 6), a timepoint at which microtubule entry into spines was inhibited. Lentivirus-driven TTL expression in these samples was performed to an extent that did not significantly affect tyrosinated/detyrosinated tubulin ratio nor spine density in control neurons (Fig. 6A–C), and we then tested for oAβ-induced spine pruning. Strikingly, in TTL-expressing neurons, oAβ completely failed to diminish spine density (Fig. 6C and D), indicating that spine loss induced by oAβ might rely on downregulation of TTL and tyrosinated tubulin levels. Global biochemical analysis showed that 100 nM oAβ did not appreciably alter the proportion of tyrosinated tubulin in these neurons (Fig. 6B). However, it was conceivable that oAβ might have locally affected the pool of tyrosinated, dynamic microtubules available for spine entry. To explore this possibility, we set out experiments to examine whether the percentage of spines invaded by dynamic microtubules correlated with spine resistance to oAβ in neurons ectopically expressing TTL (Fig. 6E–G). We found that expression of TTL averted the oAβ-induced drop in spine invasions by dynamic microtubules measured at 30 min (Fig. 6F) as well as oAβ-promoted spine loss, which became detectable only 2.5 h later (Fig. 6G). To assess whether this drop in spine entries at 30 min could be related to the loss of TTL and tyrosinated tubulin, we evaluated microtubule entries into spines when TTL levels start to decrease. In hippocampal rat neurons, after 4 days of infection with shRNA against TTL, a time point at which TTL levels begin to drop but before spine density starts to decline, microtubule entries into spines significantly decreased (Supplementary Fig. 7A and B). Accordingly, in the *TTL^+/−^* mouse neuronal cultures, there was also a significant decrease in spine entries, as compared to the wild-type (Supplementary Fig. 7C).

**Figure 7:**
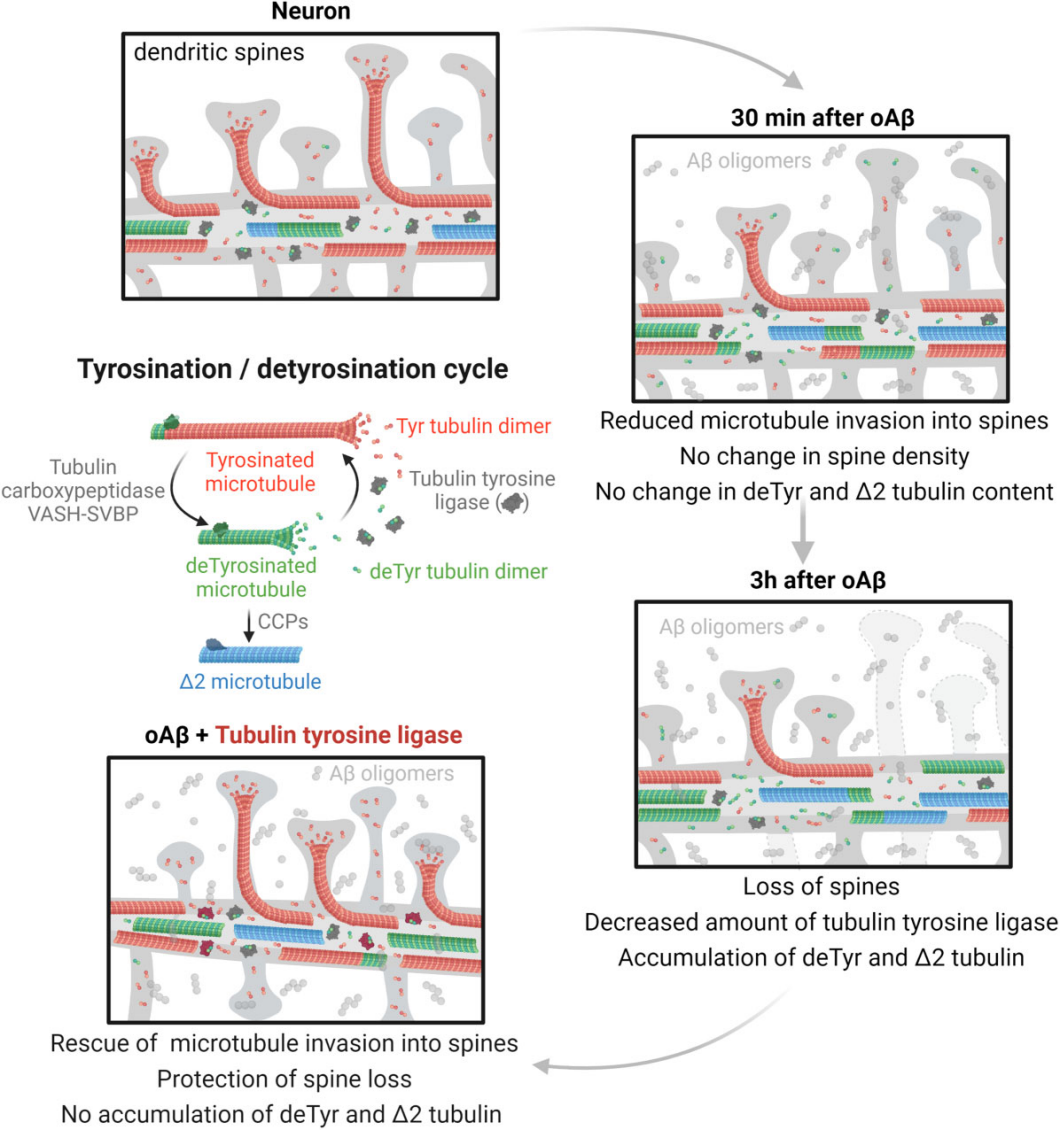
**Schematic representation of TTL, of modified tubulins in dendritic shafts and dendritic spines and of spine density in neurons (normal conditions and under oAβ exposure).** Tyrosinated tubulin dimers polymerize into dynamic tyrosinated microtubules (red). Tubulin carboxypeptidases (VASH-SVBP) detyrosinate long-lived microtubules (green). After depolymerization, TTL (in grey) retyrosinates tubulin dimers. Very stable detyrosinated microtubules are substrate of cytosolic carboxypeptidases (CCPs) to form Δ2 microtubules (blue) that exit the tyrosination/detyrosination cycle. In mature neurons from control patients (or wild-type mice), tyrosinated microtubules form a shell at the outer part of the dendrite while detyrosinated and Δ2 microtubules localize to the inner part. Some dynamic microtubules from the dendrite transiently invade dendritic spines. In neuronal models of Alzheimer’s disease, amyloid-β oligomers exposure have a sequential effect on microtubule behaviour and dendritic spine retraction: short time incubation with amyloid-β oligomers induces a decrease in TTL content, an accumulation of detyrosinated and Δ2 microtubules, a decrease in the frequency of microtubule invasion into spines with no change in dendritic spine density; longer incubation accentuates this phenotype and induces spine retraction. Ectopically controlled TTL expression restores tyrosinated, detyrosinated and Δ2 tubulin balance, microtubule invasion into the spines and dendritic spine density.

Together, our results indicate that entry of dynamic tyrosinated microtubules into spines may underlie enhanced resistance of dendritic spines to synaptic injury and that restoring TTL expression can protect dendritic spines from oAβ toxicity as illustrated in Fig. 7.

## Discussion

In this study, we identify a role for the retyrosination of α-tubulin by TTL activity in the maintenance of synaptic function and Alzheimer disease-related synaptic dysfunction.

Our biochemical and immuno-histological analysis of *TTL^+/−^* mouse hippocampi confirmed that heterozygous suppression of tubulin tyrosine ligase leads to around 40% reduction of tyrosinated tubulin, and that this reduction is compatible with viability and normal life span. This result suggests that TTL levels are rate-limiting for the maintenance of physiological amounts of tyrosinated tubulin *in vivo*.

We found that the behavioural performance of *TTL^+/−^* mice at 3 months revealed impairments in spontaneous alternation test and novel object recognition but no defect in spatial learning assessed by Morris Water Maze, the standard test for evaluating hippocampal-dependent memory in rodents. This behavioural profile was consistent with no alteration in hippocampal basal transmission and CA3/CA1 LTP at this early age, which was instead characterized by deficits in spatial working and intermediate-term recognition memory most likely caused by cortical circuitry dysfunction. In agreement with synaptic cortical damage at this age, we observed loss of dendritic spines in serial sections obtained from cortical layer V of 3-month-old *TTL^+/−^* mice. At 9 months, however, *TTL^+/−^* mice had a clear reduction in their basal hippocampal transmission, a defect consistent with decreased spine density observed in cultured hippocampal neurons from *TTL^+/−^* embryos or transiently silenced of *TTL* expression. In addition, a striking decline in the LTP of synaptic strength at the Schaffer collateral synapses was observable in 9-month-old *TTL^+/−^* mice, demonstrating that tubulin tyrosine ligase deficiency exacerbates synaptic plasticity defects with ageing.

Our in vitro analyses strongly suggest that these alterations may be related to defects in synaptic microtubule dynamics. In support of this model, we found that loss of TTL significantly reduced the number of microtubule entries into dendritic spines and led to a significant loss of synapses. In addition, we found that entry of dynamic microtubules into spines correlated with resistance to oAβ-induced spine pruning. Strikingly, expression of TTL in oAβ-treated neurons prevented both transient loss of microtubule entry into spines and spine pruning, indicating that restoring dynamic microtubule invasions into spines is the mechanism by which TTL prevents oAβ-induced loss of synapses. Matching our observed decline in TTL and tyrosinated tubulin after 30 min and 3 h of 250 nM oAβ, previous research has shown that in primary hippocampal neurons microtubules present in the dendritic shaft become less dynamic after 30 min of oAβ exposure and that detyrosinated tubulin levels increase by 3 h.^72^ The fine-tuning of the tyrosination-detyrosination tubulin cycle as a function of small, local cues may be important in the vicinity of synapses which are particularly dependent on entry of dynamic tyrosinated microtubules.^10,11^ Live imaging of spines invaded by microtubules during incubation with oAβ showed that the minority of spines that were invaded by microtubules during the recording period had a greater resistance to pruning than non-invaded spines. Given the pleiotropic effects that the tyrosination-detyrosination tubulin cycle plays in the regulation of neuronal transport,^45,94–96^ local retyrosination of tubulin by TTL might be critical for the recruitment or removal of spine modulating cargos specifically trafficked along tyrosinated microtubules. We have observed a population of spines that are not invaded by microtubules and yet persist. There may be at least two plausible explanations for this: (i) the resistant spine lacking microtubule invasion might have been invaded before video acquisition; and (ii) only a small fraction of spines is invaded at any given time, suggesting that not all spines have the same chance to be targeted by microtubules and/or are dependent on microtubules to avoid pruning. If not all spines are equally targeted, this would also explain why certain spines may be more resistant or particularly vulnerable to pruning.

Altogether, the electrophysiological, spine density and behavioural profile of *TTL^+/−^* mice shows that tubulin tyrosine ligase is required for synaptic maintenance and plasticity, and that tubulin tyrosine ligase deficiency increases synaptic vulnerability. These findings are relevant to the onset of synaptic dysfunction in neurodegenerative disease, as we find that TTL is downregulated in Alzheimer’s disease brain, human Alzheimer’s disease neurons and primary neurons exposed to oAβ. Biochemical analysis of post-mortem brain samples from clinically graded Alzheimer’s disease patients indicated a robust loss of TTL and a gain in detyrosinated and Δ2 tubulin compared to samples from non-affected individuals in the same age range. The correlation between disease conditions and non-tyrosinated tubulin accumulation was confirmed at the single neuron level by imaging analysis of Alzheimer’s disease hippocampal sections. Deficits were narrowed to an early phase of the disease, a stage at which neuron morphology appears normal with deficiencies mainly affecting the synaptic compartments. Our data also point out that in Alzheimer’s disease the accumulation of non-tyrosinated forms of tubulin affects the whole brain, suggesting that selected circuits that go awry in Alzheimer’s disease may be more vulnerable than others to loss of tubulin retyrosination.

The finding that the knock-in of the Alzheimer’s disease-linked London mutation in APP in *in vitro* differentiated human neurons also resulted in a drop in TTL compared to isogenic controls strongly supports a causal relationship between TTL loss and familial Alzheimer’s disease. Because the London mutation leads to an increase in the amyloidogenic processing of APP and overproduction of toxic amyloid-β species,^92^ the finding suggests that TTL down-regulation could be initiated by either defective APP processing and/or accumulation of oAβ. Indeed, chronic incubation of cultured mouse neurons with synthetic oAβ elicited a significant decline in TTL levels, although the underlying mechanisms are yet to be defined. The altered synaptic phenotype of *TTL^+/−^* mice suggests that downregulation of tubulin tyrosine ligase might in turn aggravate oAβ synaptoxicity by reducing microtubule dynamics, and thus cause further loss of synapses. This notion would be consistent with the protection against dendritic spine retraction that we observed in neurons in which TTL was ectopically expressed.

Altogether, our results point to a modulatory role of the tyrosination/detyrosination tubulin cycle in synaptic plasticity and indicate that loss of TTL and tubulin retyrosination are features of Alzheimer’s disease and might be one of the mechanisms playing a pathogenic role at early stages of neurodegeneration. The results also indicate that in the early stages of Alzheimer’s disease, the microtubule network appears to be less dynamic than in normal conditions, with critical loss of dynamic microtubules. They also suggest that the decrease in dynamic microtubules, rather than a global microtubule destabilization, initiates Alzheimer’s disease pathology. Our pathogenesis model does not reject loss of microtubule integrity as a major pathological feature of advanced Alzheimer’s disease, but rather proposes that amyloidogenic APP processing may affect synaptic function by reducing the population of dynamic microtubules entering into synapses at an early stage of the disease. While the molecular factors associated with the resistance of dynamic microtubules-invaded spines remain to be identified, our results indicate that TTL activators may be beneficial to restore circuit integrity in sporadic and familial Alzheimer’s disease. In addition, the VASH1/2-SVBP carboxypeptidases have been recently identified as a tubulin detyrosinating complexes^20,21^ suggesting that also drugs able to modulate tubulin carboxypeptidase activity may offer a valuable new approach for therapeutic intervention in Alzheimer’s disease.

## Supplementary Material

awab436_Supplementary_Data

## Abbreviations

fEPSPs: field excitatory postsynaptic potentials
iPSCs: induced pluripotent stem cells
LTP: long-term potentiation
oAβ: oligomeric amyloid-β peptide (1-42)
