# Supplementary material for: Tubulin tyrosination regulates synaptic function and is disrupted in Alzheimer’s disease
Source: Brain. 2022 Feb 11;145(7):2486–506. doi: 10.1093/brain/awab436 (PMC9337816; doi:10.1093/brain/awab436)
Supplement: awab436_Supplementary_Data [file awab436_supplementary_data.zip › brain-2021-01035-File009.pdf]

Supplementary materials and methods

Behavioral studies

Behavioral tests were done in 3- to 4-month-old WT and TTL$^{+/-}$ mice. Evaluation of sensorimotor function was performed by analyzing the body weight, water and food intake, muscular tone balance (tail elevation, hanging wire, pole test, chimney test and ring test), and locomotor activity. Evaluation of spatial memory was performed with Morris water maze test.

Body weight The mice were weighed with a calibrated scale, at regular intervals.

Locomotor activity Spontaneous locomotor activity was measured by using mice individually placed in Plexiglas open-field boxes (L 21.5 x W 12 x H 18 cm) equipped with infrared sensors for accurate location of the animal (Actimeter, IMETRONIC, France). The rack containing 8 boxes was connected to an electronic interface, which provides the formatting of signals from infrared sensors and allows communication with the computer. Each box remained independent and was equipped with 3 parallel infrared photocell units, all located 3 cm above the cage bottom at even intervals along the long axis of the cage, in order to assess movements within the horizontal plane. Ten additional photocell units were placed 10 cm above the cage bottom, at even intervals along the long axis of the cage, to record the frequency of rearing with which the mice stood on their hind legs in the field (vertical activity). Locomotor activity was assessed by counting the number of interruptions of the horizontal photocell units located 3 cm above the cage bottom. This parameter was automatically computed. Spontaneous locomotor activity was recorded in 10 min intervals for 60 min.

Rotarod The rotarod test is used to assess motor coordination, ataxia and balance in young adult male mice. Mice had to keep their balance on a rotating rod. The time (latency) taken by the mouse to fall down the rod was measured. In the morning of test day, mice were trained to walk on the rotating rod (rotarod apparatus, LETICA, BioSeb) under continuous speed of 40 rpm during 5 minutes. During the training session, in the afternoon the mice were put back in the rotating rod with an increasing speed (from 4 to 40 rpm).

Water and food intake The mice were placed in a cage, with the same stall as the supplier. The food and water were weighed before the animals are brought together. After 24 h or 3 days, the food and water were weighed again.

Muscular tone balance

Tail elevation test The mice was suspended by the tail and the spontaneous position of the limbs was observed during 5 seconds. The normal position is with limbs in an escaped extended position with no clasping whereas abnormal position is with limbs entirely retracted and touching the abdomen (scores between 5 and 0).

Wire hang test: the mice were placed in the center of the sieve about 10 cm above a table. The sieve is slowly turned over and the falling latency is recorded during 3 tests lasting a maximum of 2 minutes with 10–15 s intervals.

Pole test The mice were placed at the top of a vertical wooden pole (height 60 cm) and the ability of the mice to grasp, to descend to the base of the pole was evaluated in 3 consecutive trials. Behavior is ranked normal when the mice perfectly climbing down with 4 paws, intermediate when the mice is climbing down with difficulties and abnormal when the mice do not grip the test pole (scores between 5 and 0).

Chimney test The mice were introduced head forward in a Pyrex glass cylinder (3 cm diameter and 30 cm length) held in a horizontal position. The tube is then moved to a vertical position and the ability of the mice to climb backwards out was monitored. Behavior is ranked normal when the mice use the 4 legs and reach the top of the tube quickly, intermediate when the mice reach the top of the tube with difficulty and abnormal when the mice cannot go backwards (scores between 5 and 0).

Ring test The mice were put on a metal ring and the locomotor behavior was monitored. Behavior is ranked normal when the mice perfectly climb and grip the ring with 4 paws, intermediate when the mice grip with difficulties and abnormal when the mice do not grip the ring (scores between 5 and 0).

Morris water maze test This was conducted in a pool filled with white tinted water that contain a submerged platform always placed at the same place 1 cm below the surface of the water. During learning, mice are subjected to several tests in the pool, in which they must learn, starting from different locations (N, S, E and W), to swim to the platform guided by marks available outside the pool. After 24 hours, the mice are subjected to a retention test to evaluate their spatial memory. The percentage of time spent in the goal quadrant was measured for each animal the mean and standard errors of the mean (SEM) were calculated for each group.

Plasmids

The cDNAs encoding LifeAct (aa 1-17 of *Saccharomyces cerevisiae* ABP140, NP_014882) C-terminally fused to RFP, and encoding human EB3 (NP_001289979) C-terminally fused to YFP

were cloned in pLV-mCherry vector (Addgene #36084, kind gift from Didier Trono). During the cloning process, mCherry cDNA was removed and replaced by an IRES (encephalomyocarditis virus internal ribosome entry site) sequence to allow EB3-YFP expression. PCR amplification and cloning of cDNAs was performed with Phusion DNA polymerase (Thermo Scientific) and In-Fusion HD Cloning kit (Clontech), respectively. The construct was verified by sequencing (Eurofins and Genewiz) and purified with HiPure Plasmid Maxiprep kits (Invitrogen).

Lentivirus production

Lentiviral particles were produced using the second-generation packaging system. Lentivirus encoding both LifeAct-RFP and EB3-YFP (cloned in a PLV-mCherry derived vector) were produced by co-transfection with the psPAX2 and pCMV-VSV-G helper plasmids (Addgene plasmids # 12260 and # 8454, gifts from Didier Trono and Bob Weinberg, respectively), into HEK293T cells (ATCC-CRL-3216) using the calcium phosphate transfection method. Viral particles were collected 48 h after transfection by ultra-speed centrifugation, prior to aliquoting and storage at -80°C.

Biochemical analysis of cultured primary rat neurons

Primary hippocampal neurons isolated from rat embryos were transduced or not with tubulin tyrosine ligase lentivirus. For the oAβ experiment, neurons were transduced at DIV14 expressing tubulin tyrosine ligase and treated with 250nM oAβ for 30 minutes or 3 hours. For the knockdown of tubulin tyrosine ligase, neurons were transduced with a control or 1 of 2 independent tubulin tyrosine ligase-targeting shRNAs. Lysates were collected and western blotting was performed in the same manner as described for the iPSC cells in the main manuscript.

Microtubule invasion into spines in TTL KD and TTL +/- neurons

Rat hippocampal neurons from WT rat embryos were infected at DIV 17 with lentiviral vectors containing either control or shTTL1, a tubulin tyrosine ligase-targeting shRNA, and incubated until DIV 21. Neurons were then co-transfected with plasmids encoding EB3-eGFP and tdTomato using Lipofectamine 2000 (Invitrogen) 48h before performing live cell imaging. Imaging and analysis of microtubule entries into spines were performed as described in the live imaging of microtubule dynamics at spines section in the main manuscript.

Mouse hippocampal neurons from WT and $TTL^{+/-}$ embryos were grown on 35 mm glass bottom live imaging dishes (ibidi) and infected at 10 DIV with a lentivirus containing LifeAct-RFP and EB3-YFP cDNA. Live cell imaging was performed at 18 DIV using an inverted microscope (Axio Observer, Zeiss) coupled to a spinning-disk confocal system (CSU-W1-T3, Yokogawa) connected to a wide-field electron-multiplying charge-coupled device (CCD) camera (ProEM+1024, Princeton Instrument) with a 63×/1.46 oil objective and maintained at 37°C and 5% CO2. Movies of microtubule dynamics at spines were acquired at 5 second/frame for 10 minutes with 4 z-stack planes at 0.7 µm step size by Metamorph software. Maximum projections of movies were performed in ImageJ and % of spines invaded in 10 minutes were quantified as described in the main manuscript.

Supplemental figures

Figure S1

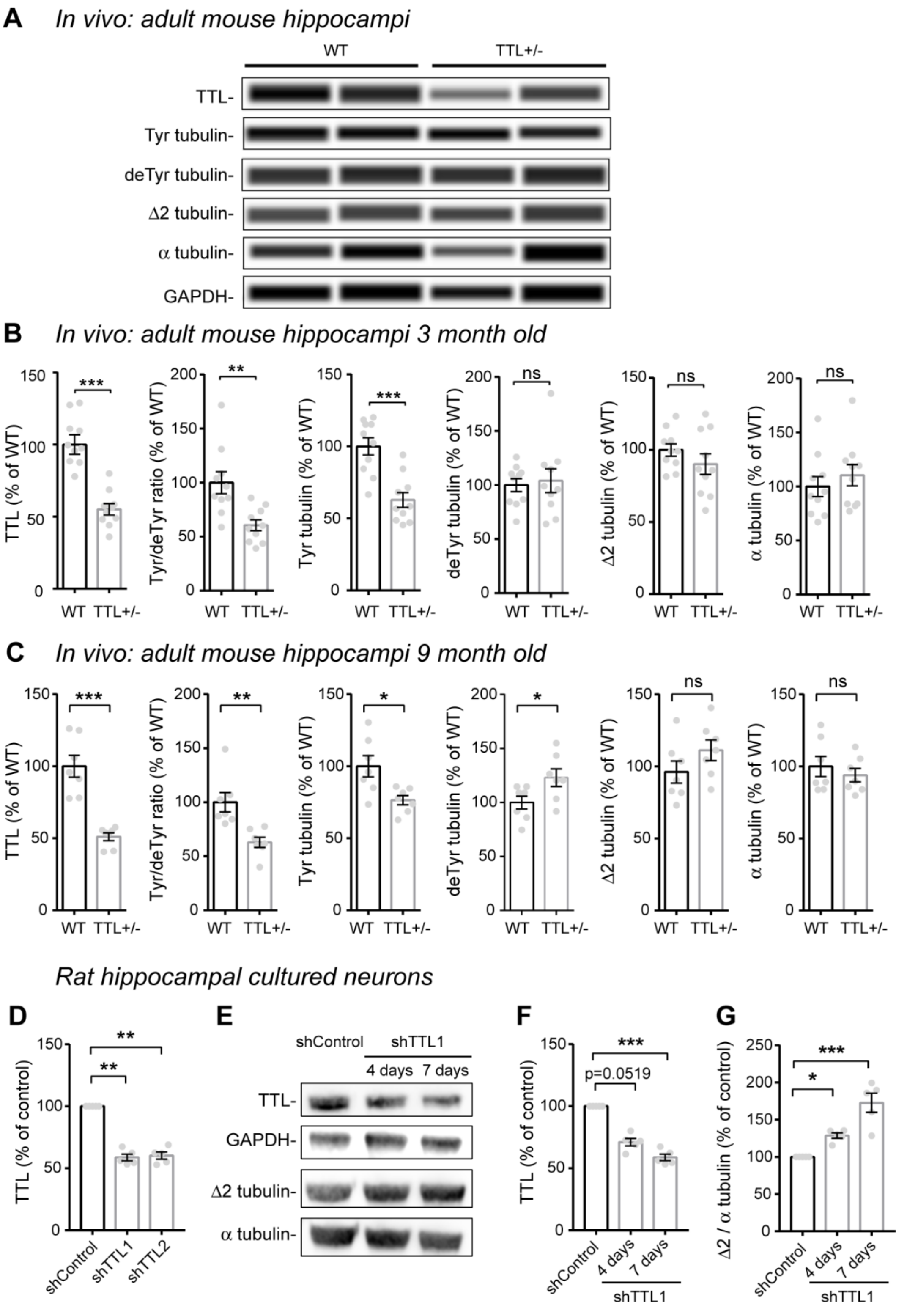

Supplementary Figure 1. Analysis of tubulin tyrosine ligase levels and α-tubulin modifications linked to the tubulin tyrosination/detyrosination cycle in protein extracts

from WT and tubulin tyrosine ligase heterozygous hippocampi and in WT neurons silenced of tubulin tyrosine ligase expression. (**A-C**) PeggySue analysis of tubulin tyrosine ligase (TTL) and selected modified tubulin levels in 3 and 9-month-old WT and tubulin tyrosine ligase heterozygous ($TTL^{+/-}$) mouse hippocampi (samples preparation and analysis as in main text, Fig 1B). (A) Examples of protein expression levels. (**B-C**) Quantification of relative amounts of proteins. All values are normalized with GAPDH and expressed as % of WT. Graphs represent mean ± SEM. n = 10 and 7 in 3- and 9-month-old mice groups, respectively. Mann-Whitney's test *** $p < 0.001$. (**D**) Evaluation of shRNA efficacy by quantification of relative amounts of tubulin tyrosine ligase in cultured rat hippocampal neurons infected with control shRNA or with 2 shRNA lentivirus targeting tubulin tyrosine ligase (shTTL 1 and shTTL 2) during 7 days. All values are normalized with GAPDH and expressed as % of control. Graphs represent mean ± SEM. $n = 5$ cultures for each condition. Kruskal-Wallis with Dunn's multi-comparison test, *** $p < 0.01$. (**E**) Immunoblot analysis of tubulin tyrosine ligase (TTL), GAPDH, Δ2 and α-tubulin levels in cultured rat hippocampal neurons infected with control shRNA (non-coding) or shRNA lentivirus targeting tubulin tyrosine ligase (shTTL 1) for 4 days and 7 days. Quantification of relative amounts of TTL normalized with GAPDH (**F**) and Δ2 tubulin normalized to total tubulin (**G**), expressed as % of control. $n = 5$ cultures for each condition. Kruskal-Wallis with Dunn's multi-comparison test, $p = 0.0519$ and *** $p < 0.001$.

Figure S2

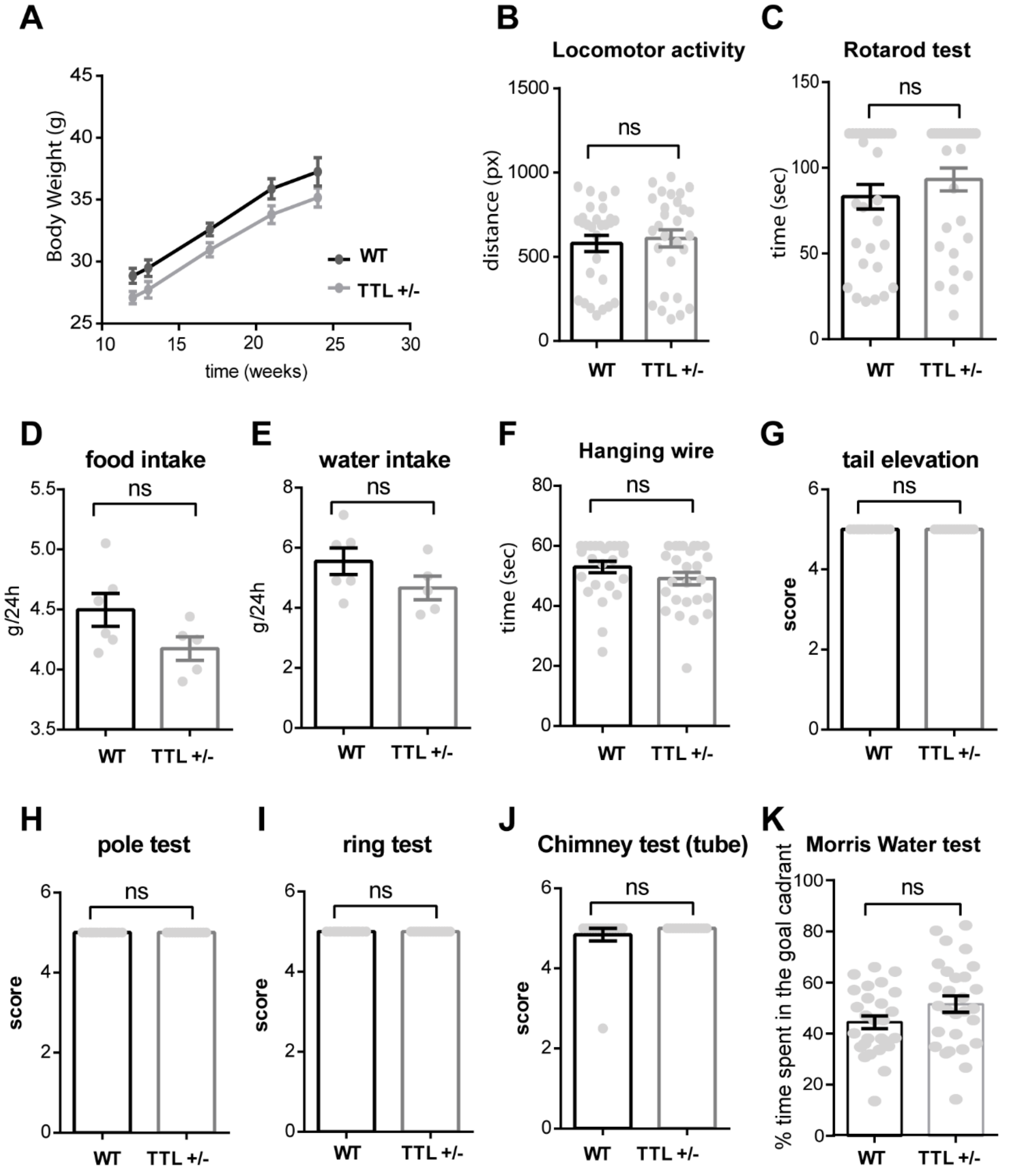

Supplementary Figure 2: Normal sensorimotor function, locomotor activity and spatial memory of tubulin tyrosine ligase heterozygous mice. (**A**) Body weight of WT and tubulin tyrosine ligase heterozygous ($TTL^{+/-}$) mice was measured in function of time. Two Way ANOVA, genotype x time interaction (F (4, 88) = 0.0681, p = 0.9914). n = 12 WT and $TTL^{+/-}$ mice. Source of variation: genotype, * p = 0.020. (**B**) Spontaneous locomotor activity was analyzed by measuring total distance covered by WT and $TTL^{+/-}$ mice in open field boxes in 30 minutes. n = 28 WT and $TTL^{+/-}$ mice. Student t test, ns = not significant. (**C**) Motor coordination was analyzed by rotarod test. The time taken by the mouse to fall down the rod was measured

for WT and TTL$^{+/-}$ mice. *n* = 30 WT and TTL$^{+/-}$ mice. Student t test, ns = not significant (**D-E**). Water and food intake were measured by weighing water and food at the beginning of the test and 24 hours later, for WT and TTL +/- mice. *n* = 6 WT and 5 TTL$^{+/-}$ mice. Student t test, ns = not significant. **(F-J)** Fine motor coordination was analyzed by various tests: (F) Hanging wire test (*n* = 26 WT and TTL$^{+/-}$ mice, Student t test, ns = not significant), (G) Tail elevation, (H) Pole test, (I) Ring test and (J) Chimney test (*n*=16 WT and 15 TTL$^{+/-}$ mice. Mann-Whitney test, ns = not significant). (**K**) Evaluation of spatial memory was performed with Morris water maze test. The percentage of time spent in the goal quadrant was measured for WT and TTL$^{+/-}$ mice. n = 27 and 28 for WT and TTL$^{+/-}$ mice. Student t test, ns = not significant. (**B-K**) All graph represents mean ± SEM.

Figure S3

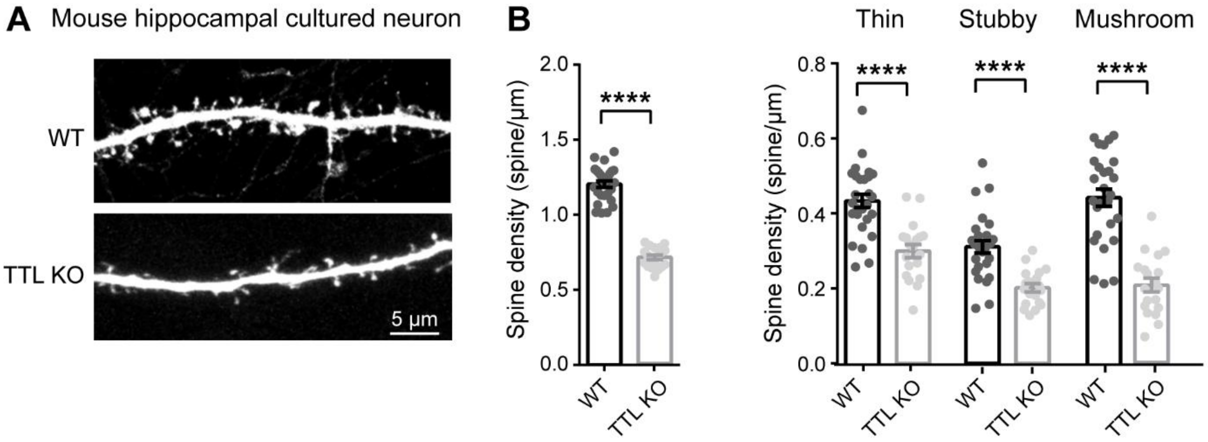

Supplementary Figure 3. Total tubulin tyrosine ligase suppression results in dramatic loss of dendritic spine density. (A) Confocal images showing representative examples of dendritic segments of GFP-expressing WT and tubulin tyrosine ligase knock-out (TTL KO) hippocampal neurons in culture at 17 DIV. **(B)** Total dendritic spine density, or that of each different morphological type of spines are represented from WT and TTL KO hippocampal cultured neurons. Graphs represent mean ± SEM. *n* = 27 and 19 neurons from WT and TTL KO embryos from at least 3 independent cultures. Student's t test. **** $p<0.0001$.

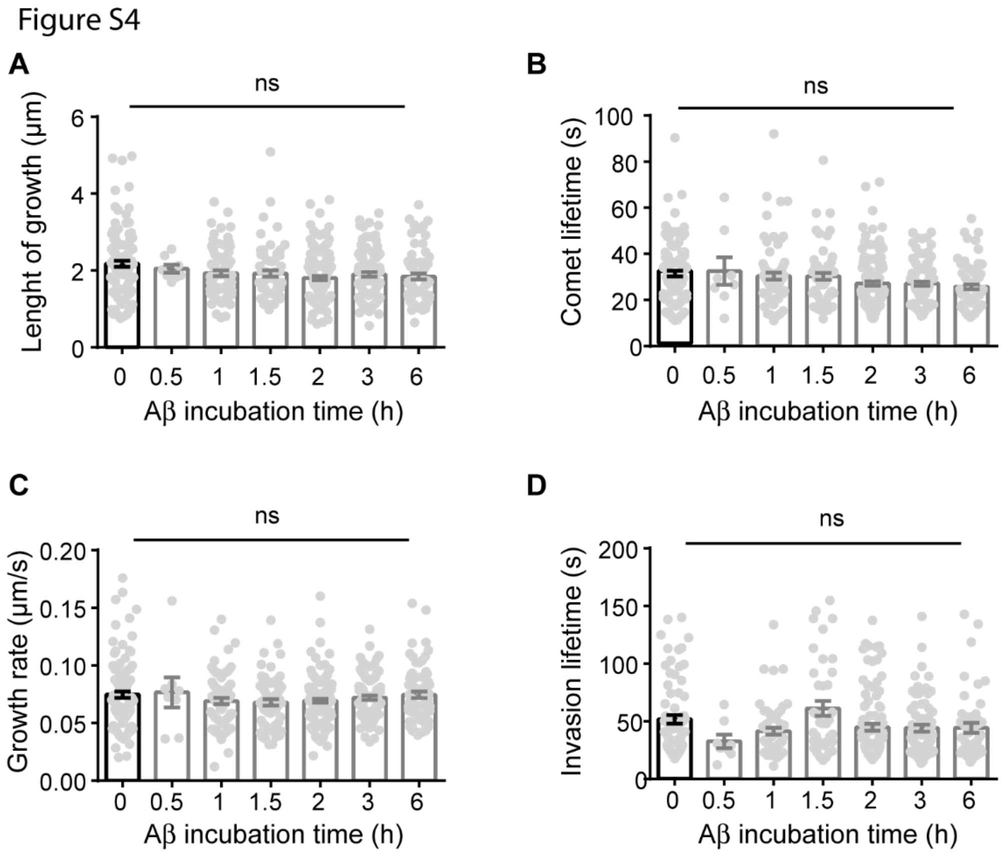

Supplementary Figure 4. Dynamic parameters of microtubules invading spines before and after oAβ treatment. Comet length growth (**A**), comet lifetime (**B**), microtubule growth rate (**C**) and invasion lifetime (**D**) during 10-minute movies in WT hippocampal neurons expressing EB3-EGFP and DsRed before and after oligomeric amyloid β peptide (1-42) (oA β) (250 nM) incubation at the indicated times. Graphs represent mean ± SEM. *n*=112, 8, 76, 66, 138, 104, and 73 comets were analyzed at each time point, respectively. Kruskal-Wallis test, ns= not significant.

Figure S5

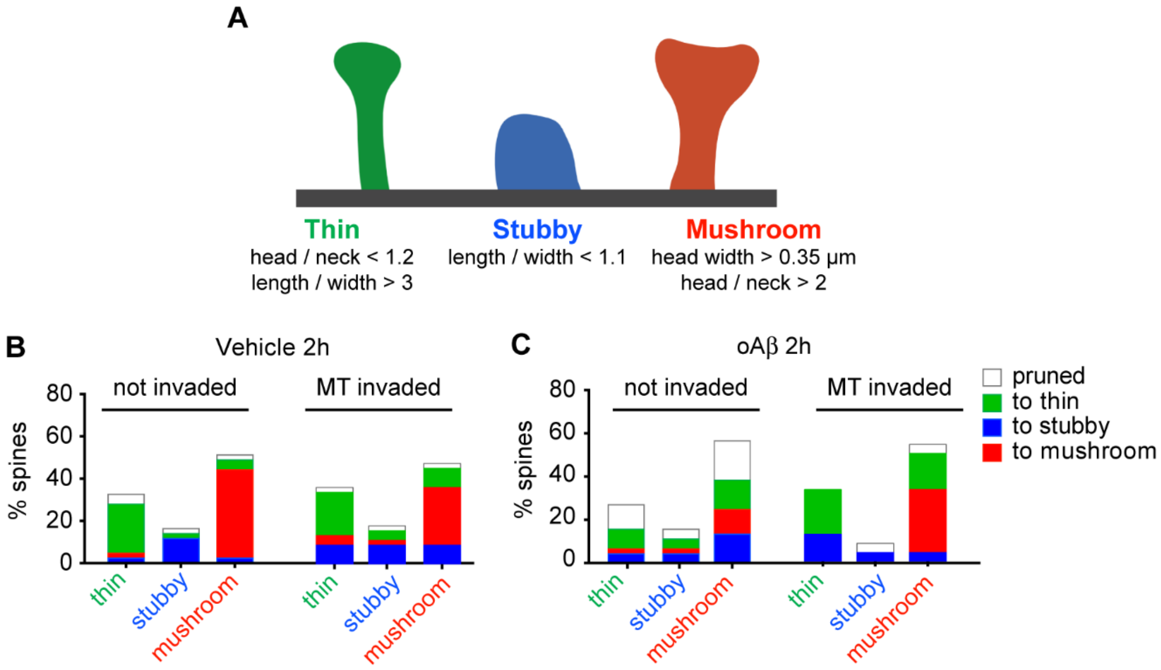

Supplementary Figure 5. Structural plasticity of spines invaded or not by dynamic microtubules. (**A**) The following ratios were used to identify thin, stubby and mushroom spines in Neurolucida and NeuronStudio: thin spines were identified as having a head / neck ratio of < 1.2 and length / width ratio of > 3. Spines were considered stubby if they had a length to width ratio of < 1.1. Finally, mushroom spines were typed as having a head width > 0.35 µm and a head to neck ratio of > 2. **(B-C**) Morphologies (stubby, mushroom, thin) of all dendritic protrusions invaded or not invaded by EB3-labeled growing microtubule plus ends before and after vehicle (B) or 250 nM oAβ treatment (C) were individually documented. Percentages of the same protrusions changing to pruned, thin, mushroom, or stubby spines were then calculated based on total number of spines invaded or not invaded by EB3 in the same field. The X^2 test was performed to determine whether the 4 different conditions (Vehicle 2 hours not invaded, Vehicle 2 hours microtubule invaded, oAβ 2 hours not-invaded and oAβ 2 hours microtubule-invaded) were associated with significantly different proportions of the 4 possible morphological outcomes (to stubby, to mushroom, to thin, to pruned). Overall dependence of spine fate on the four conditions was significant (X^2 = 53.98, 9 df, p = < .0001).

Figure S 6

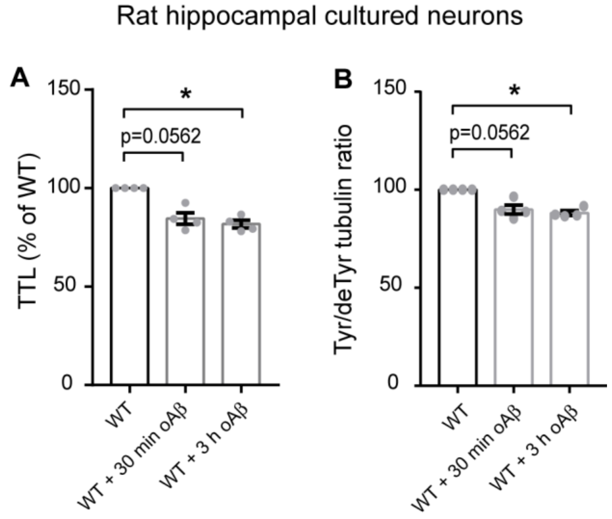

Supplementary Figure 6: Time course effect of oAβ treatment on tubulin tyrosine ligase and modified tubulins accumulation in rat hippocampal neurons. (A-B) Immunoblot analysis of tubulin tyrosine ligase (TTL) content (**A**) and of tyrosinated/detyrosinated tubulin ratio (**B**) from WT rat hippocampal neurons (17 DIV) treated with DMSO or with 250 nM oAβ, for 30 minutes or for 3 h. Data are expressed as a % of WT and graphs represent mean ± SEM. (**A**) n = 4 cultures for WT, WT+ 30 min oAβ and WT+ 3h oAβ respectively. Kruskal-Wallis with Dunn's multi-comparison test, $p = 0.0562$ and * $p < 0.05$. (**B**) n = 4 cultures for WT, WT+ 30 min oAβ and WT+ 3h oAβ respectively. Kruskal-Wallis with Dunn's multi-comparison test, $p = 0.0562$ and * $p < 0.05$.

Figure S7

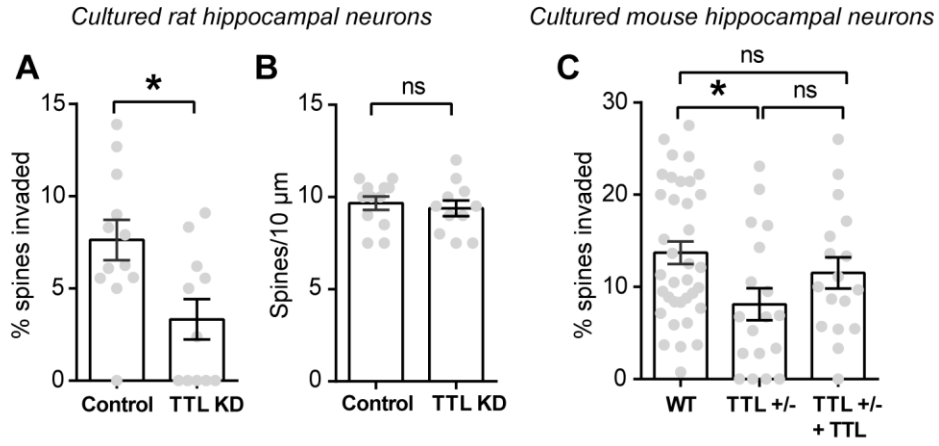

Supplementary Figure 7. Effect of tubulin tyrosine ligase on microtubule invaded spines. Percentage of spines invaded by microtubules (**A**) and dendritic spine density (**B**) of rat hippocampal neurons infected with control shRNA or with shRNA lentiviruses targeting tubulin tyrosine ligase for 4 days prior to analysis (TTL KD). Graphs represent mean ± SEM. (**A**) n = 12 and 11 of control and TTL KD neurons respectively. Student t test, * $p < 0.05$. Number of spines analyzed: 273 and 240 for WT and TTL KD, respectively. (**B**) n =12 and 11 for control and TTL KD neurons respectively. Student t test, ns = not significant. (**C**) Percentage of spines invaded by microtubules in WT and TTL+/- mouse hippocampal neurons (18 DIV) transduced or not with a lentivirus containing TTL cDNA. n =36, 18 and 17 for WT, TTL+/- and TTL+/- +TTL neurons, respectively. One Way ANOVA with Sidak's multi comparison test. * $p < 0.05$ and ns = not significant. Number of spines analyzed: 836, 700 and 478 for WT, TTL+/- and TTL+/- +TTL neurons, respectively.

Table S1

Control patients				
Patient Number		Gender	Age	Cause of death
#61	Control	male	65	pulmonary embolism, arterosclerosis, heart failure
#199	Control	female	76	heart failure
#223	Control	male	61	heart failure
#220	Control	male	63	pulmonary embolism
#164	Control	male	85	acute cardiorespiratoric insufficiency
#198	Control	female	68	parieto-occipital and frontal stroke, pneumonia, respiratory insufficiency
#205	Control	male	66	cardiovascular-pulmonary insufficiency, acute lymphoid leukemia
#217	Control	male	60	acute myocardial infarction
#266	Control	male	61	vertebrobasilar stroke (right side)
#269	Control	male	79	stroke in the left cortical hemisphere, herniatio
#276	Control	female	79	stroke, pneumonia
**Alzheimer's disease patients**				
#184	Braak stadium I - II	male	83	emolitio cerebri
#187	Braak stadium I - II	male	62	respiratory and cardiac insufficiency
#191	Braak stadium I - II	female	93	Alzheimer's disease, cardiovascular-respiratory insufficiency
#218	Braak stadium I - II	female	72	acute cardiac insufficiency
#219	Braak stadium I - II	male	67	pulmonary embolism
#185	Braak stadium III - IV	male	80	stroke (right side), herniatio
#196	Braak stadium III - IV	female	78	stroke, arteria cerebri media, brain hemorrhage
#230	Braak stadium III - IV	female	79	pulmonary embolism
#197	Braak stadium III - IV	male	64	myocardial infarction
#267	Braak stadium III - IV	female	91	stroke, arteria cerebri media (left side)
#279	Braak stadium III - IV	male	79	stroke (right side)
#154	Braak stadium V-VI	female	72	acute myocardial infarction, earlier heart failure, arterosclerosis
#167	Braak stadium V-VI	female	65	suicide (hanging - asphyxia)
#195	Braak stadium V-VI	male	83	respiratory and cardiac insufficiency
#202	Braak stadium V-VI	male	84	cardiac and respiratory insufficiency
#212	Braak stadium V-VI	female	87	dementia, myocardial insufficiency
#229	Braak stadium V-VI	female	78	Alzheimer's disease
#232	Braak stadium V-VI	female	77	cardiorespiratory insufficiency

Table S1. Details of control and Alzheimer's disease patients classified into Braak stages as shown in Figure 3A-E. The gender, age and cause of death are described for each patient. As the post-mortem interval (PMI) between the time of death and the collection of tissues is a critical factor affecting the quality of human brain tissues, we analyzed only samples with a PMI inferior of 300 minutes.

Table S2

Post#	MPN08-17	MPN03-166	MPN09-270	MPN07-47	OC04-18	MPN03-161
Case & Classification	Case 2 Control	Case 3 Control	Case 4 Control	Case 9 AD	Case10 AD	Case 11 AD
Age	74	87	90	75	82	86
Sex	M	M	M	M	M	M
Braak NFT stage	3	3	3	6	6	6
CERAD plaque score	none	sparse	sparse	frequent	frequent	frequent
Amyloid angiopathy	0	0	0	++	++	++
NIAR	0	Low	Low	High	High	High
PMI (min)	93	310	270	295	127	260

Table S2. Table of case descriptions of postmortem human Alzheimer's disease and control brains shown in Figure 3F-H. The age and gender are listed for each patient. The Braak NFT stage, CERAD plaque score and amyloid angiopathy are listed to demonstrate the pathological hallmarks of Alzheimer Disease present in each brain sample. The NIA-Reagan score (NIAR) is a post-mortem diagnosis score of the likelihood of having Alzheimer's disease, which considers the Braak stage and CERAD score. We analyzed only samples with a PMI less than or close to 300 minutes.
