# Supplementary material for: Tubulin tyrosination regulates synaptic function and is disrupted in Alzheimer’s disease
Source: Brain. 2022 Feb 11;145(7):2486–506. doi: 10.1093/brain/awab436 (PMC9337816; doi:10.1093/brain/awab436)

Full unedited blots fig 3A

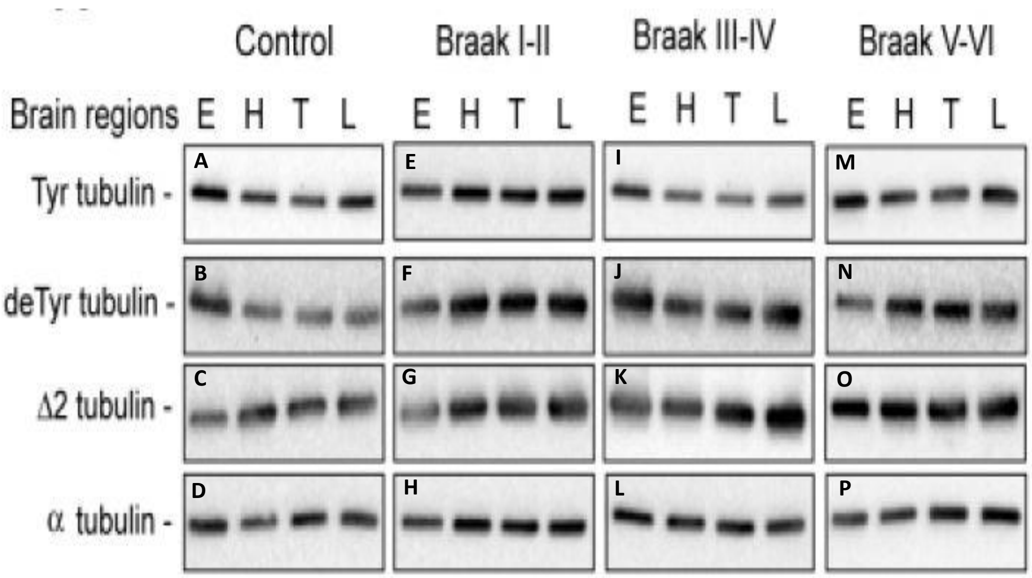

Control

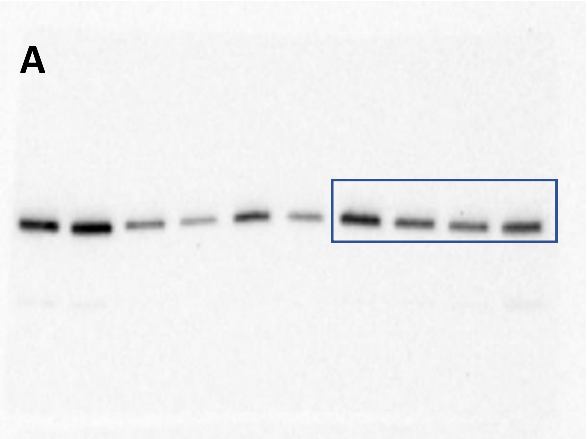

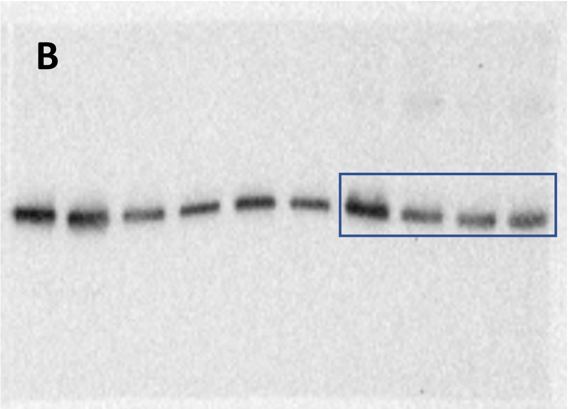

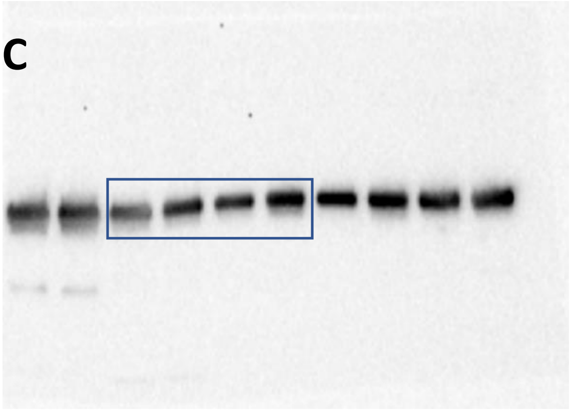

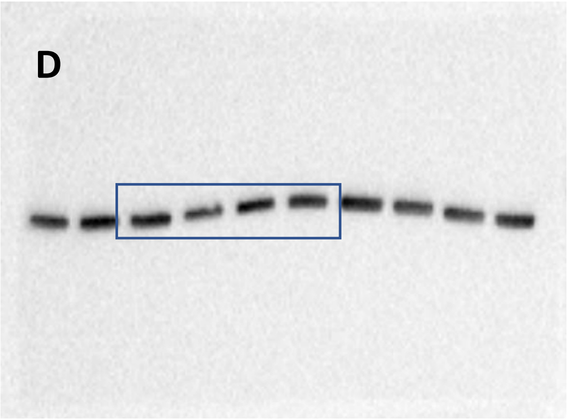

Full unedited blots fig 3A

Braak I-II

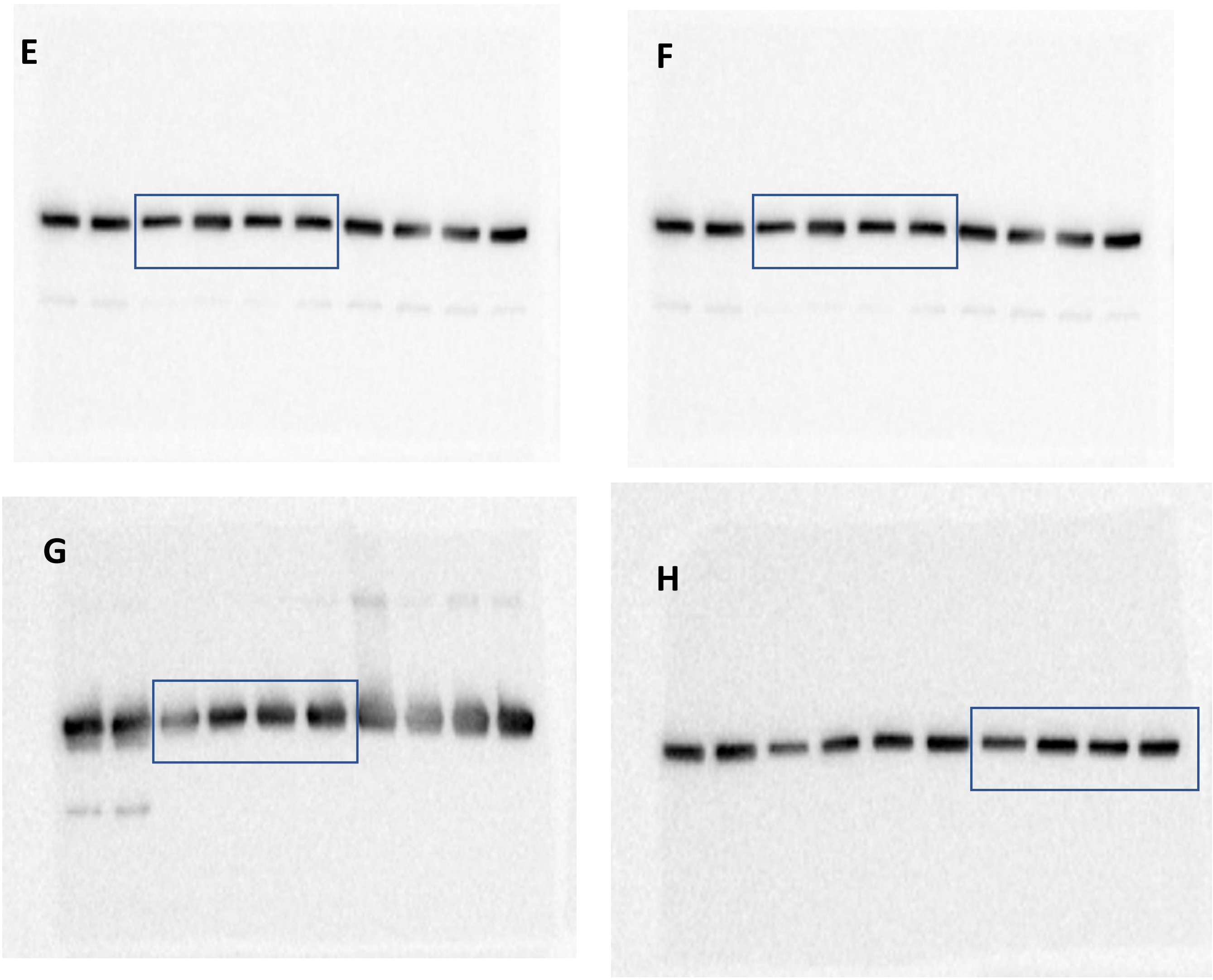

Full unedited blots fig 3A

Braak III-IV

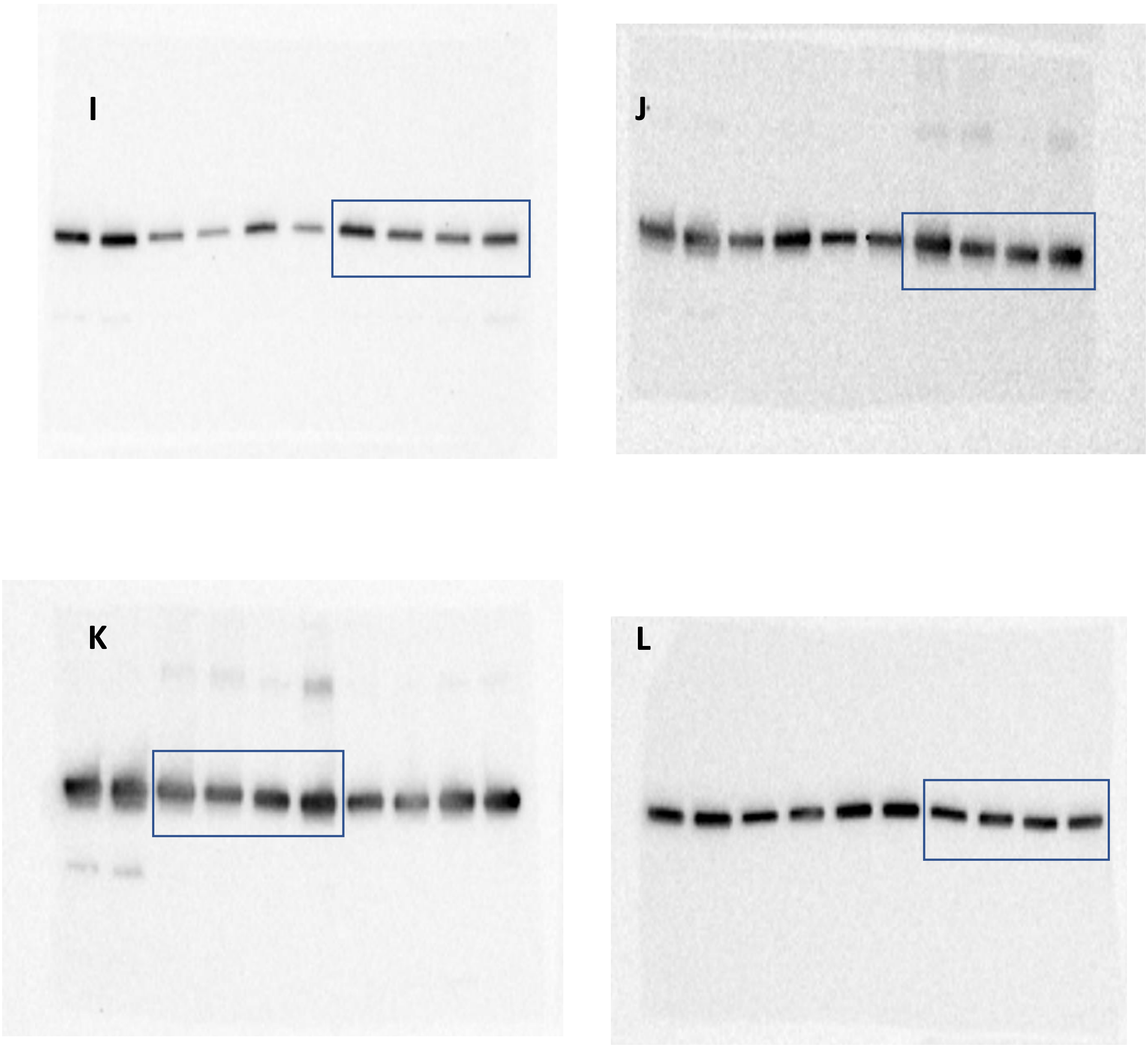

Full unedited blots fig 3A

Braak V-VI

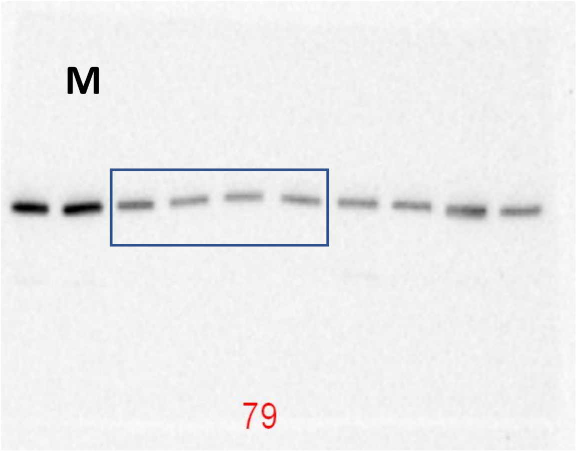

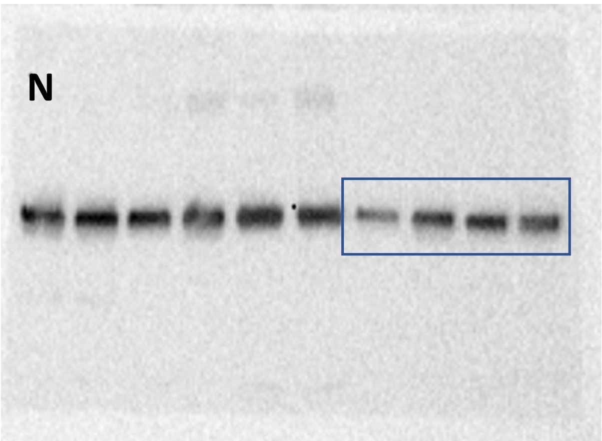

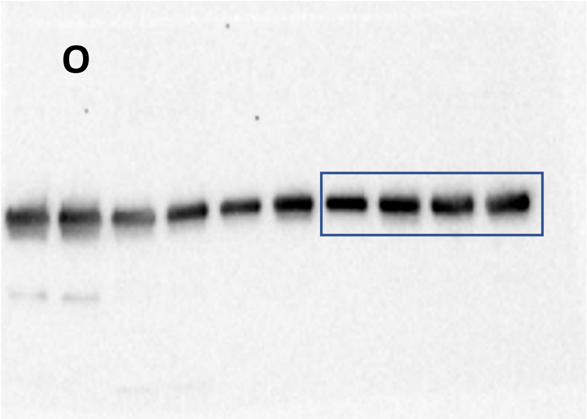

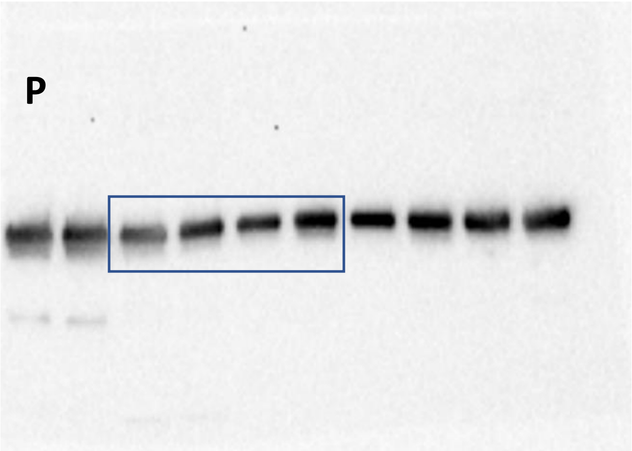

Full, unedited blots: Figure 4A

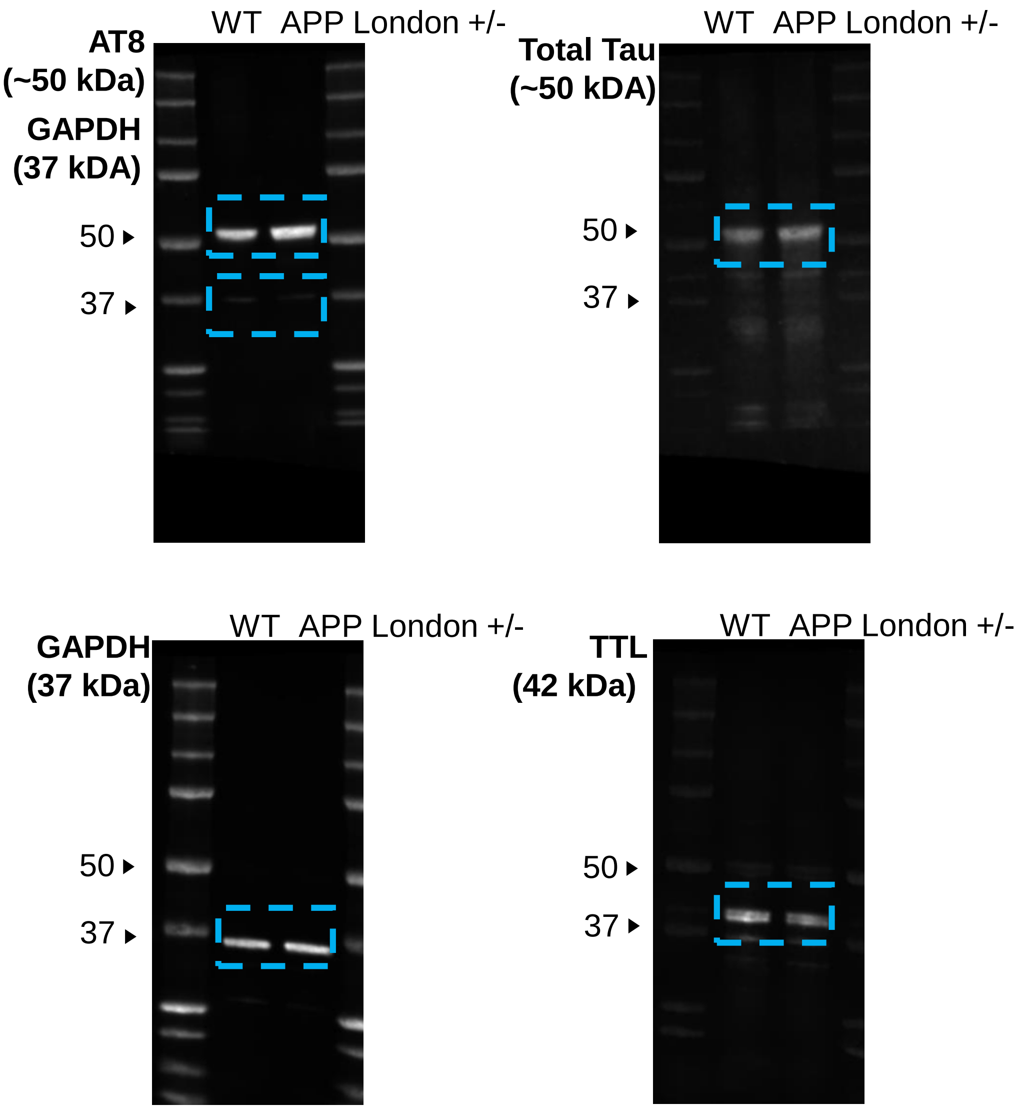

Full, unedited blots: Figure 4A Continued

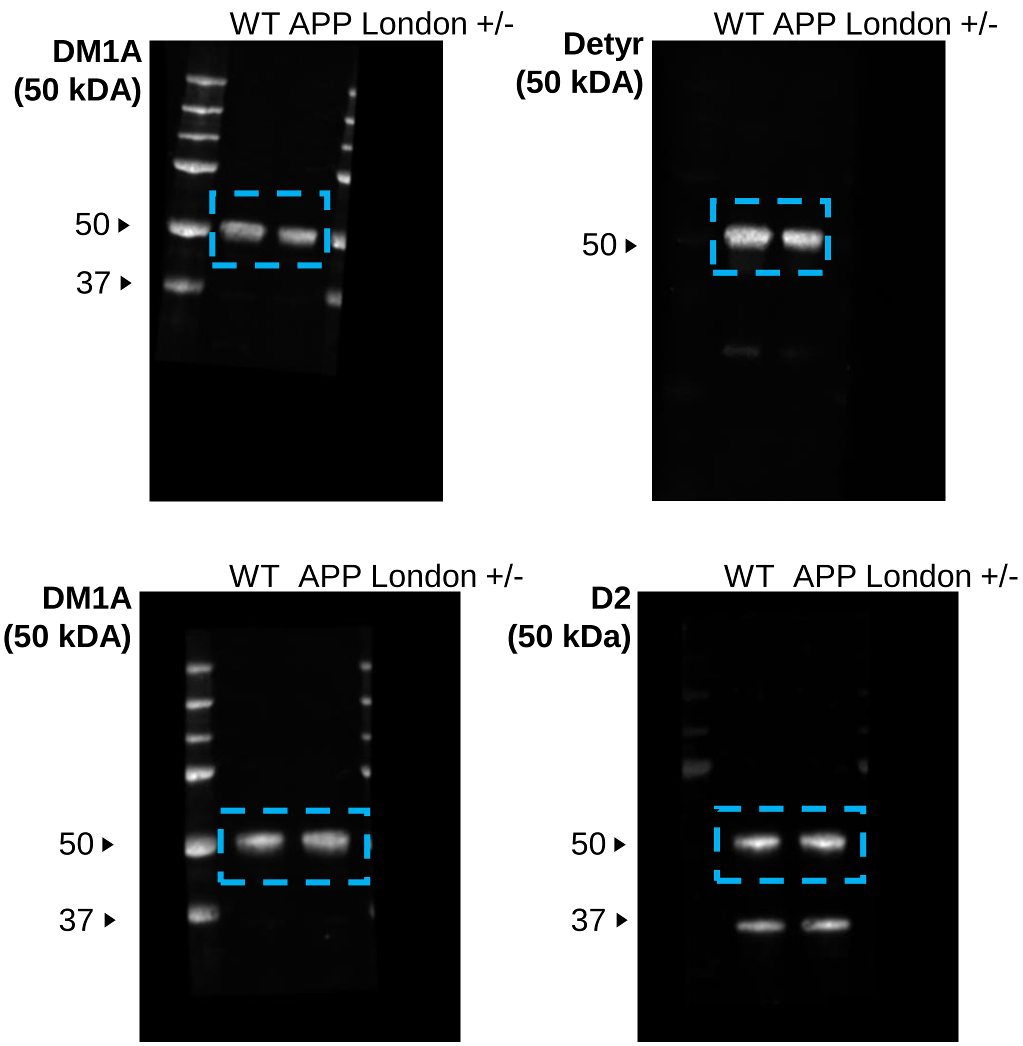

Full, unedited blots: Figure S1E

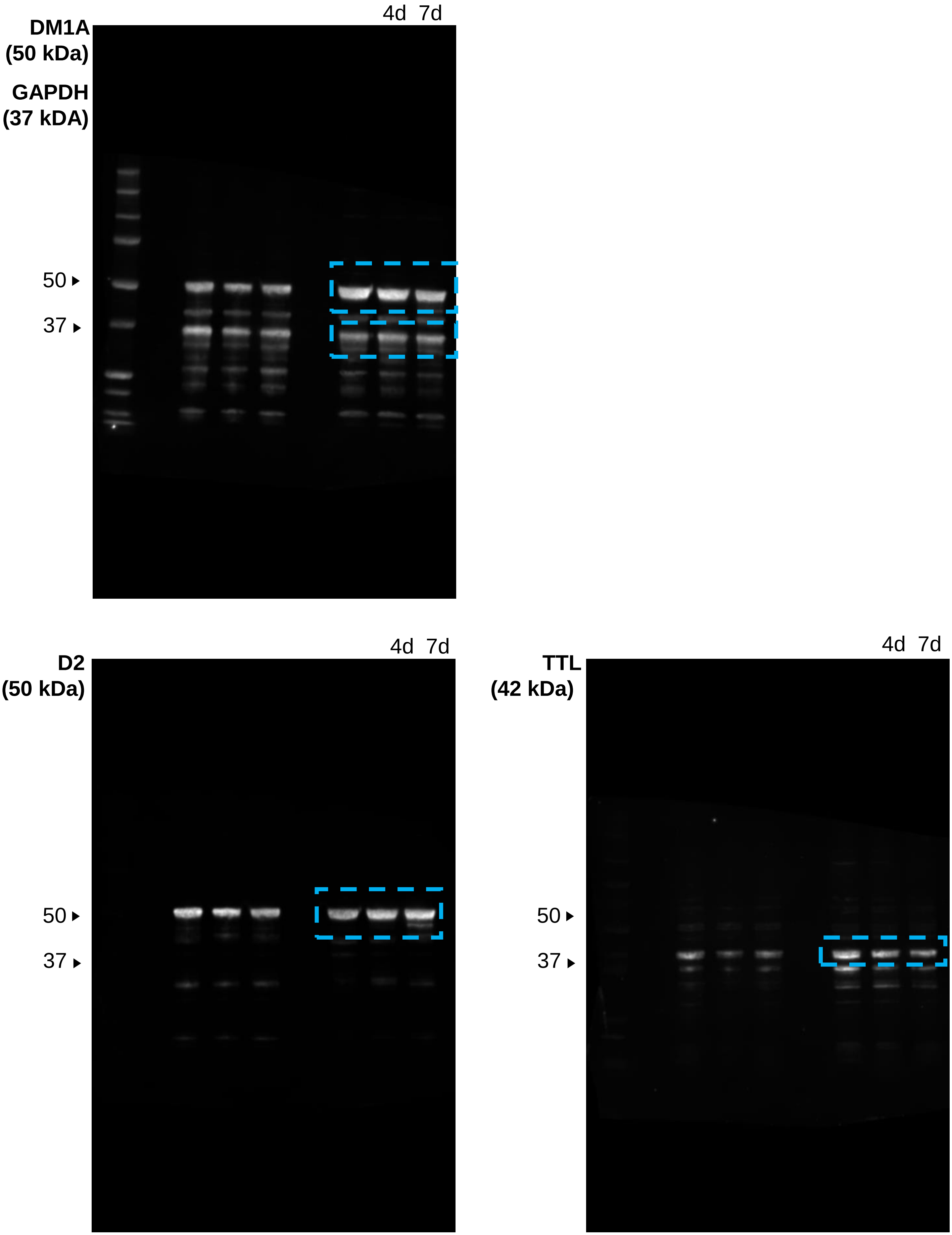

Supplement: awab436_Supplementary_Data [file awab436_supplementary_data.zip › brain-2021-01035-File010.pdf]
